# Genome-wide analysis of UDP-glycosyltransferases family and identification of UGT genes involved in abiotic stress and flavonol biosynthesis in *Nicotiana tabacum*

**DOI:** 10.1186/s12870-023-04208-9

**Published:** 2023-04-19

**Authors:** Qing Yang, Yinchao Zhang, Xiaoling Qu, Fengyan Wu, Xiuchun Li, Min Ren, Ying Tong, Xiuming Wu, Aiguo Yang, Yong Chen, Shuai Chen

**Affiliations:** 1 Tobacco Research Institute, Chinese Academy of Agricultural Sciences, Qingdao, 266101 China; 2 Qujing Tobacco Company of Yunnan Province, Qujing, 655000 China; 3 China National Tobacco Corporation, Beijing, 100045 China

**Keywords:** UDP-glycosyltransferase, *Nicotiana tabacum*, Expression pattern, Abiotic stress, Flavonol glucosides

## Abstract

**Background:**

Uridine disphosphate (UDP) glycosyltransferases (UGTs) act upon a huge variety of highly diverse and complex substrates, such as phytohormones and specialized metabolites, to regulate plant growth, development, disease resistance, and environmental interactions. However, a comprehensive investigation of UGT genes in tobacco has not been conducted.

**Results:**

In this study, we carried out a genome-wide analysis of family-1 UDP glycosyltransferases in *Nicotiana tabacum*. We predicted 276 *NtUGT* genes, which were classified into 18 major phylogenetic subgroups. The *NtUGT* genes were invariably distributed among all the 24 chromosomes with structural diversity in exon/intron structure, conserved motifs, and *cis*-acting elements of promoters. Three groups of proteins which involved in flavonoid biosynthesis, plant growth and development, transportation and modification were identified that interact with NtUGT proteins using the PPI analysis. Expression analysis of *NtUGT* genes in cold stress, drought stress and different flower color using both online RNA-Seq data and the realtime PCR analysis, suggested the distinct role of *NtUGT* genes in resistance of cold, drought and in flavonoid biosynthesis. The enzymatic activities of seven NtUGT proteins that potentially involved in flavonoid glycosylation were analyzed, and found that all seven exhibited activity on myricetin; six (NtUGT108, NtUGT123, NtUGT141, NtUGT155, NtUGT179, and NtUGT195) showed activity on cyanidin; and three (NtUGT108, NtUGT195, and NtUGT217) were active on the flavonol aglycones kaempferol and quercetin, which catalyzing the substrates (myricetin, cyanidin or flavonol) to form new products. We further investigated the enzymatic products and enzymatic properties of NtUGT108, NtUGT195, and NtUGT217, suggested their diverse enzymatic activity toward flavonol, and NtUGT217 showed the highest catalyzed efficient toward quercetin. Overexpression of *NtUGT217* significantly increase the content levels of the quercetin-3-O-glucoside, quercetin-3-O-rutinoside and kaempferol-3-O-rutinoside in transgenic tobacco leaves.

**Conclusion:**

We identified 276 *UGT* genes in *Nicotiana tabacum*. Our study uncovered valuable information about the phylogenetic structure, distribution, genomic characters, expression patterns and enzymatic activity of *NtUGT* genes in tobacco. We further identified three *NtUGT* genes involved in flavonoid biosynthesis, and overexpressed *NtUGT217* to validate its function in catalyze quercetin. The results provide key candidate *NtUGT* genes for future breeding of cold and drought resistance and for potential metabolic engineering of flavonoid compounds.

**Supplementary Information:**

The online version contains supplementary material available at 10.1186/s12870-023-04208-9.

## Background

Glycosylation is an important modification reaction that plays crucial roles in plant growth and in responses to biotic and abiotic stresses [1]. Glycosyltransferases (GTs) facilitate glycosylation by catalyzing the transfer of sugar molecules from activated donors to specific receptors [2]. Family-1 GTs, usually referred to as uridine disphosphate (UDP) glycosyltransferases (UGTs), are the most common GTs in plants and have significant effects on plant growth and development. UGT proteins possess a highly conserved consensus sequence near the C-terminal that is 44 amino acids (aa) in length and is referred to as the plant secondary product glycosyltransferase (PSPG) box [3]. An increasing number of putative UGT-encoding genes have been identified in plants, including 107 in *Arabidopsis thaliana*, 130 in *Prunus mume*, 147 in maize, 179 in wheat, 180 in rice, 181 in grape, 182 in soybean, and 241 in apple [4-9]. Diverse multi-gene UGT family members function together to modulate complicated biochemical processes in plant cells, which in turn affects numerous biological activities and functions.

UGT proteins catalyze the conversion of sugar groups from UDP activated sugars to substrates such as hormones and specialized metabolites, and are therefore involved in biosynthesis of natural plant products such as flavonoids, terpenoids, steroids, and hormones, which regulate plant growth, development [10, 11]. However, only a few UGT proteins have been documented. For example, 26 UGT proteins from the UGT71, UGT73, UGT74, UGT85, UGT91, and UGT94 families in multiple plants have been functionally characterized as catalyzing triterpene glycosylation [12]. *UGT79B1* and *UGT91A1* in *Arabidopsis*, strawberry, peach, and kiwifruit were reported to mediate anthocyanin modification [13-15]. *UGT13*, *UGT72AD1*, *UGT72AF1*, *UGT72AH1*, *UGT72V3*, *UGT72Z2*, *UGT73A20/24/25*, *UGT73C20*, *UGT73F2*, *UGT76F1*, *UGT78A14*, *UGT78D1/2/3*, *UGT80B1*, *UGT80A2*, *UGT88A1*, *UGT88E14/15/16/18/19*, *UGT92G4*, *UGT91Q2*, and *UGT716A1* have been isolated and confirmed to function in catalyzing flavonoid glycosylation [16-21]. In *Arabidopsis*, 118 *UGT* genes were shown to be differentially expressed in response to treatment with 2,4-dichlorophenoxyacetic acid and trichostatin, abscisic acid (ABA) and salicylic acid (SA), indole acetic acid (IAA), methyl jasmonate (MeJA), or zeatin [11]. *UGT71B6*, *UGT71B7*, and *UGT71B8* play crucial roles in ABA homeostasis and in adaptation to various abiotic stresses [22]. *UGT84B1* and *UGT74D1* modulate IAA levels throughout plant development by dual IAA and oxIAA glycosylation [23]. *UGT72AD1* and *UGT72Z2* overexpression results in significant inhibition of soybean root growth, suggesting a role of these genes in auxin homeostasis [19].

UGTs not only function as glycosylate acceptor molecules, but also play pivotal roles in biotic and abiotic stress responses. They function in stabilizing and enhancing water solubility, inactivating or detoxifying natural products [24], promoting regulation of metabolic homeostasis and detoxifying exogenous substances [3]. *TaUGT2*, *TaUGT3*, *TaUGT4*, and *TaUGT1287* were reported associated with scab resistance [25, 26]. Down-regulation of *CsUGT91Q2* and *CsUGT78A14* reduces cold-stress tolerance [27, 28]. *AtUGT76b1* knockout mutants have a dwarf phenotype and a constitutive *Pseudomonas* infection defense response [29]. In rice, *UGT90A1* and *IAGT1* are significantly up-regulated in response to a combined hydroxyurea and IAA treatment, suggesting that these genes activate auxin-glucose conjugation to protect rice seedlings against hydroxyurea-induced phytotoxicity [30]. In this study, we aimed to identify putative *UGT* genes in tobacco and to further explore *NtUGT* genes that may be involved in abiotic stress resistance and flavonoid biosynthesis. We also aimed to characterize the enzymatic properties of recombinant NtUGT proteins to provide a basis for further functional analyses in the future.

## Results

### Identification of* NtUGT *genes in* N. tabacum*

Complete sequencing and de novo genome assembly of the common tobacco cultivar K326 has greatly facilitated the identification of tobacco gene families. Using the latest release of the tobacco genome, we used HMMER 3.0 and the HMM profile of the conserved UGT domain to search *N. tabacum* for putative *UGT* genes. After removing genes encoding redundant proteins and sequences that were too short or too divergent, 276 candidate UGT proteins were identified for further analysis (GenBank accession numbers: OP616116-OP616391). All candidates were between 98 and 1443 aa in length and contained the signature PSPG motif. All of the identified UGTs contained two major domains, a conserved C-terminal domain and a variable N-terminal domain, although the overall sequence diversity was high between genes. The *NtUGT* genes were named based on their chromosomal location (or scaffold location, where necessary). The predicted subcellular locations of the *NtUGT* genes varied widely, although most were predicted to be localized to the chloroplast (129). Seventy-five *NtUGTs* were predicted to be located in the cytoplasm, and 37 were predicted to be located in the nucleus (Supplementary Table S4 and Figure S1).

GO term analysis of all 276 putative *NtUGT* genes showed that they were associated with 15 molecular function terms and 23 biological process terms (Supplementary Figure S2). Most of the putative *NtUGT* genes were annotated as being involved in flavonoid biosynthesis, flavonoid metabolic processes, and various flavonoid glucuronidation processes (Supplementary Figure S2A). The putative *NtUGT* genes were associated with 20 major KEGG biochemical pathways related to general and specialized metabolic processes (Supplementary Figure S2B and Table S5), including starch and sucrose metabolism, sphingolipid metabolism, ether lipid metabolism, flavonoid biosynthesis, phenylpropanoid biosynthesis, carotenoid biosynthesis, steroid hormone biosynthesis, zeatin biosynthesis, ascorbate and aldarate metabolism, drug metabolism, retinol metabolism, chemical carcinogenesis, metabolism of xenobiotics by cytochrome P450, porphyrin and chlorophyll metabolism, glucosinolate biosynthesis, cyanoamino acid metabolism, pentose and glucuronate interconversions, and tryptophan metabolism.

### Phylogenetic analysis of NtUGT proteins

**Fig. 1:**
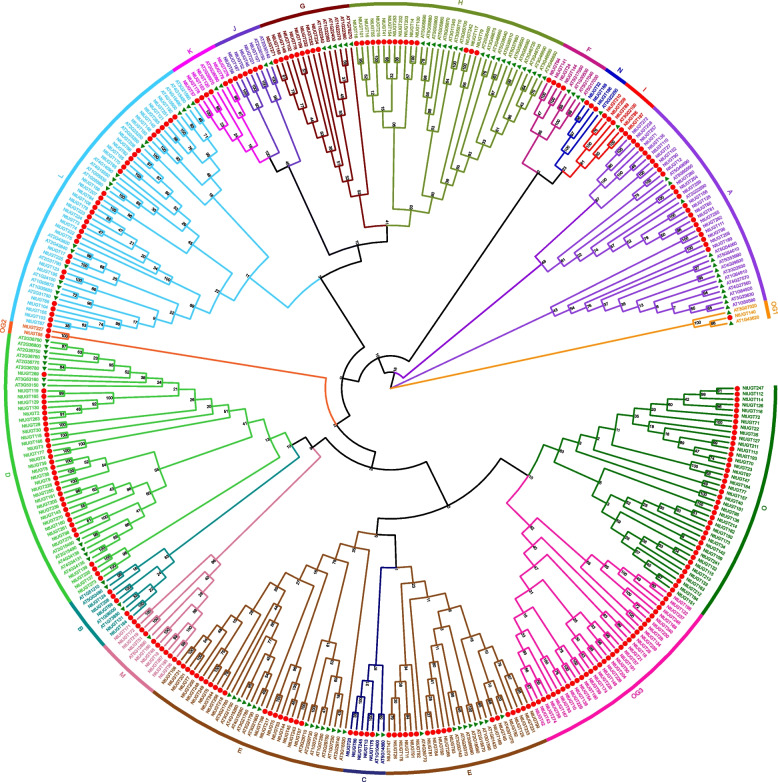
Phylogenetic analysis of UGT family members in *Nicotiana tabacum*. ClustalW and MEGA7 were used for alignment of the full-length sequences of 276 tobacco and 114 Arabidopsis UGTs and for phylogenetic tree construction. A green triangle at the end of a branch indicates an Arabidopsis gene whereas a red circle indicates a tobacco gene

The relationships between the 276 tobacco and 114 *Arabidopsis* UGT proteins identified were next analyzed. Based on the protein sequences of typical *Arabidopsis* UGTs in each group, the phylogenetic tree constructed from these 390 proteins showed 18 major subgroups, namely 14 subgroups (A–N) that were present in all higher plants [4, 5], one O subgroup that was present in some higher plant species such as maize [32], and three out-group subgroups (OG1-OG3), which not belong to present known subgroups. (Fig. 1). Subgroup O had the most members (40 proteins), followed by subgroup E (37 proteins). Statistical analysis of the distribution of NtUGT protein functions showed that nearly all proteins with the same function were present in the same subgroup. As reported UGTs in *Arabidopsis, Brassica* species, maize and soybean, UGT proteins related to polyphenolic and flavonoid synthesis always enriched in subgroup A, E, F, L and M [4, 11, 13-18], proteins related to terpene metabolism were enriched in subgroups D and M [12, 28], proteins related to plant hormones were enriched in subgroups H, L, K, and O [11, 19, 22, 23], and proteins related to zeatin metabolism were almost enriched in subgroup O [5].

### Chromosomal distribution of* NtUGT *genes

Using the annotation information for *NtUGT* genes from the *N. tabacum* genome database, the chromosomal locations of *NtUGT* genes were illustrated to determine *NtUGT* distribution in tobacco. Due to the relatively low assembly quality of the *N. tabacum* genome, only 123 *NtUGT* genes could be mapped to chromosomes. The mapped *NtUGT* genes were unevenly distributed across 24 chromosomes. The number of *NtUGT* genes varied between 1 and 15 per chromosome, with relatively high density observed on chromosomes 17, 13, 15, and 24 (Supplementary Figure S3). All *NtUGT* genes located in chromosome 17 were members of subgroup E. Tandem duplication events were also analyzed to determine the importance of duplication events in shaping chromosomes and causing tandem exons in *NtUGT* genes. To analyze the syntenic relationships of *NtUGTs*, we mapped the 18 pairs of *NtUGTs* derived from tandem duplication to the duplicated blocks (Supplementary Figure S3). Eighteen gene pairs among 35 *NtUGT* genes were derived from tandem duplication. Interestingly, gene pairs derived from tandem duplication were each present in the same subgroup, and most of the gene pairs had the same function (Table 1). This suggested that tandem duplication may have played a major role in expansion of the *NtUGT* gene family. *Ka/Ks* analysis revealed that the 18 pairs of genes were under selective pressure; eight were under positive selection, whereas the other 10 pairs were under purifying selection (Table 2).

**Table 1:** Distribution of *NtUGT* gene pairs derived from tandem duplication

**Genes**	**Chromosome**	**Subgroup**	**Functions**
NtUGT60&NtUGT61	13	A	UDPglycosyltransferase91A1like
NtUGT3&NtUGT4	2	D	UDP-glycosyltransferase 73C4/C6-like
NtUGT8&NtUGT9	2	D	scopoletin glucosyltransferase-like
NtUGT38&NtUGT39	10	D	UDP-glucose flavonoid 3-O-glucosyltransferase 7-like; scopoletin glucosyltransferase-like
NtUGT118&NtUGT119	24	D	UDP-glycosyltransferase 73D1/C1-like
NtUGT6&NtUGT7	2	E	UDP-glycosyltransferase 71E1-like; anthocyanidin 3-O-glucosyltransferase 2-like
NtUGT44&NtUGT45	11	E	UDP-glucose flavonoid 3-O-glucosyltransferase 6/2-like
NtUGT91&NtUGT92	17	E	anthocyanidin 3-O-glucosyltransferase 5-like
NtUGT80&NtUGT81	17	E	anthocyanidin 3-O-glucosyltransferase 5-like
NtUGT82&NtUGT83	17	E	UDP-glycosyltransferase 72D1-like; anthocyanidin 3-O-glucosyltransferase 5-like
NtUGT15&NtUGT16	4	H	7-deoxyloganetic acid glucosyltransferase-like
NtUGT67&NtUGT68	15	L	UDP-glycosyltransferase 74E2-like
NtUGT64&NtUGT65	15	L	crocetin glucosyltransferase, chloroplastic-like
NtUGT65&NtUGT66	15	L	crocetin glucosyltransferase, chloroplastic-like
NtUGT73&NtUGT74	15	L	UDP-glycosyltransferase 74G1-like
NtUGT22&NtUGT23	5	O	zeatin O-glucosyltransferase-like
NtUGT113&NtUGT114	23	O	zeatin O-glucosyltransferase-like
NtUGT57&NtUGT58	13	OG3	beta-D-glucosyl crocetin beta-1,6-glucosyltransferase-like

**Table 2:** Ka/Ks analysis of tandem duplicated gene pairs

**Sequence**	**Method**	**Ka**	**Ks**	**Ka/Ks**
NtUGT38&NtUGT39	MA	0.530023	0.740223	0.716031
NtUGT91&NtUGT92	MA	0.335388	0.174309	1.92411
NtUGT64&NtUGT65	MA	0.839818	0.388703	2.16057
NtUGT65&NtUGT66	MA	0.328493	3.94315	0.083307
NtUGT3&NtUGT4	MA	0.143144	0.816305	0.175356
NtUGT22&NtUGT23	MA	0.314315	2.11433	0.14866
NtUGT67&NtUGT68	MA	0.219034	0.180018	1.21674
NtUGT44&NtUGT45	MA	0.171685	0.093027	1.84555
NtUGT15&NtUGT16	MA	0.195502	0.801498	0.243921
NtUGT6&NtUGT7	MA	0.581701	0.501098	1.16085
NtUGT8&NtUGT9	MA	0.403736	2.98856	0.135094
NtUGT60&NtUGT61	MA	0.530274	0.374227	1.41698
NtUGT73&NtUGT74	MA	0.074043	0.193997	0.38167
NtUGT80&NtUGT81	MA	0.081176	0.050937	1.59366
NtUGT113&NtUGT114	MA	0.437107	0.829576	0.526904
NtUGT118&NtUGT119	MA	0.570816	2.29355	0.248879
NtUGT57&NtUGT58	MA	0.468377	0.936109	0.500345
NtUGT82&NtUGT83	MA	0.434823	0.339281	1.2816

### Genomic characteristics of *NtUGT* genes

We analyzed the exon/intron structure, conserved motifs, and *cis*-acting elements in *NtUGT* promoters to investigate the structural diversity among these genes. We found that exon number was not evenly distributed. There were 109 *NtUGT* genes with no introns and 161 with at least one intron. Of the 161 intron-containing *NtUGT* genes, 115 possessed one intron, 33 had two introns, and the remaining genes possessed between three and 16 introns (Supplementary Figure S4). *NtUGT140* had the largest number of exons and the most complex gene structure, with 17 exons and 16 introns. In the phylogenetic tree, most *NtUGT*s with the same number of introns clustered together. Most of the intron-less *NtUGT*s were clustered in subgroups O (22 intron-less *NtUGT*s), OG3 (20), D (20), E (13), and A (11). *NtUGT*s with single intron were primarily clustered in subgroups L, E, O, H, A, G, OG3, and D, the latter of which contained 26 single-intron genes. *NtUGT* genes with two introns were mainly clustered in subgroups E (seven), OG3 (six), and D (five). In general, members of each subgroup exhibited similarity in intron/exon characteristics (Supplementary table S6).

**Fig. 2:**
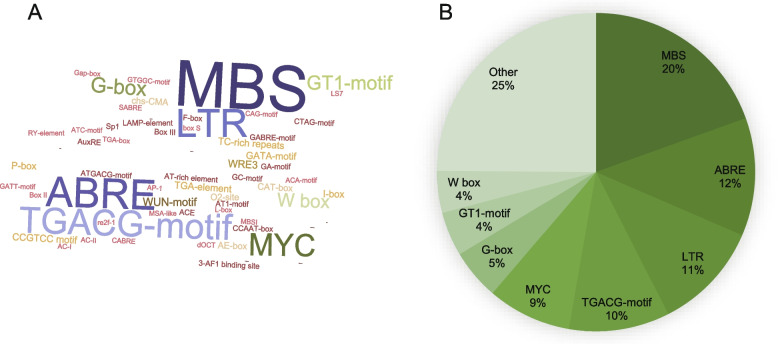
Types and numbers of *cis*-acting elements in *NtUGT* promoters. **A** There were 4472 *cis*-elements of 63 different types identified in *NtUGT* promoters, including MBSs, ABREs, MYCs, LTRs, and GT1s. **B** Total and relative abundance of different types of *cis*-elements in *NtUGT* promoters

During the long evolutionary history of plants, gene expression has been precisely regulated by transcription factors. *Cis*-elements in gene promoter sequences determine which transcription factors bind to a gene to regulate expression. In this study, we conducted a detailed investigation of all *cis*-elements present in the promoter regions (classified as 1500 bp upstream of the transcription start site) of *UGT* family genes. We found a total of 4472 *cis*-elements of 63 different types (Fig. 2A). The most abundant were MYB-binding sites (MBSs) (20%), ABREs (12%), LTRs (11%), TGACG-motifs (10%), MYCs (9%), G-boxes (5%), GT1-motifs (4%) and W boxes (4%) (Fig. 2B). MBSs were associated with drought stress, light responsiveness, and flavonoid biosynthesis regulation. ABREs were involved in responses to abiotic stress and light. G-boxes and GT1-motifs were involved in light responsiveness; LTRs were associated with low-temperature responsiveness; MYCs were involved in light responsiveness and temperature responsiveness; TGACG-motifs were involved in MeJA responsiveness; and W boxes were associated with SA responsiveness. We also identified other *cis*-elements that were associated with responses to gibberellin (namely the TATC-box, GARE-motif, and P-box), auxin (the TGA-element), and light (the GATA-motif, MRE, TCT-motif, ATCT-motif, chs-CMA1a, chs-CMA2a, and ACE). These results indicated that *NtUGT* gene expression may be influenced by a wide range of developmental processes and environmental factors.

To further analyze the distribution of major *cis*-elements in *NtUGT* genes, the type and number of *cis*-elements in each subgroup were counted and described in detail (Supplementary Figure S5). MBSs were the most abundant type of *cis*-element in most of the subgroups, except in subgroups B, C, and F; in those three subgroups, the most abundant types were ABREs, G-boxes, and MYCs, respectively. Subgroup E contained the largest number of *cis*-element types (50), followed by subgroups L and O (49 each).

### NtUGT protein–protein interaction (PPI) networks

**Fig. 3:**
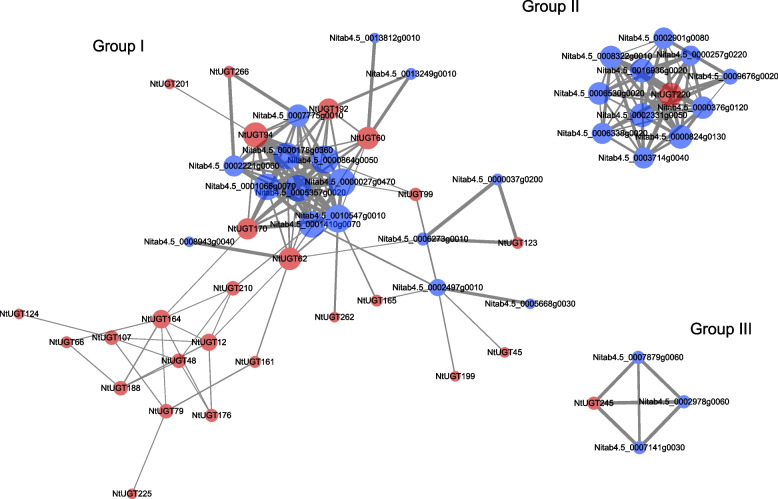
NtUGT protein–protein interaction (PPI) network. Each node in the PPI network represents all proteins generated by the associated single gene. Node size represents the degree of interaction and edge thickness represents the strength of the interaction between two proteins. Nodes representing NtUGTs are red whereas those representing non-NtUGT proteins that interact with NtUGTs are blue

Mining the proteins that interact with NtUGTs can aid in understanding NtUGT functions. Using NtUGTs as bait proteins revealed a total of three groups of interacting proteins. Group I comprised 25 NtUGTs that interacted with each other (red nodes) and with other types of proteins (blue nodes) (Fig. 3). NtUGT60, NtUGT94, NtUGT62, NtUGT170, and NtUGT192 were found to interact with five flavonoid biosynthesis related structure genes, including Nitab4.5_0000027g0470 (chalcone isomerase-like, CHI), Nitab4.5_0001066g0070 (chalcone synthase, CHS), Nitab4.5_0001410g0070 (flavonoid 3'-monooxygenase), Nitab4.5_0010547g0010 (leucoanthocyanidin dioxygenase, LDOX), Nitab4.5_0000178g0360 (dihydroflavonol 4-reductase, DFR), and Nitab4.5_0005357g0020 (anthocyanidin synthase, ANS),which suggested that NtUGT60, NtUGT94, NtUGT62, NtUGT170, and NtUGT192 may be involved in flavonoid metabolism and have important roles in flavonoid glycosylation. Meanwhile, NtUGT60 and NtUGT192 were found also interact with Nitab4.5_0013249g0010 (CYP77B1), a fatty acid epoxygenase specific to flowering plant [33]. NtUGT94 was found also interact with Nitab4.5_0002221g0060 (ferulate-5-hydroxylase, F5H) and Nitab4.5_0007775g0010 (caffeic acid 3-O-methyltransferase, COMT), which was important structure genes involved in lignin biosynthesis. NtUGT60, NtUGT192 and NtUGT94 may be function in both flavonoid biosynthesis and in other metabolism. The results suggested that some NtUGTs have broad substrates and function in various biological process. In Group II, NtUGT220 was found to interact with 11 proteins, including Nitab4.5_0006530g0020 (pentatricopeptide repeat protein, PPR), Nitab4.5_0008322g0010 (RNA binding domain of NusB), Nitab4.5_0009676g0020 (homeobox transcription factor, ISS), Nitab4.5_0002331g0050 (ribonuclease III C terminal domain), Nitab4.5_0002901g0080 (transcription-repair coupling factor), Nitab4.5_0016936g0020 (RNA recognition motif) and Nitab4.5_0006338g0020 (which contains a DNA-binding motif found in homing endonucleases), which were associated with DNA replication, transcription, and translation. We therefore hypothesized that NtUGT220 may play an important role in plant growth and development. In Group III, NtUGT245 was found to interact with three proteins: Nitab4.5_0002978g0060 (potassium transporter), Nitab4.5_0007141g0030 (serine/threonine-protein kinase fray), and Nitab4.5_0007879g0060 (protein furry homolog-like). These proteins may be associated with the function of transportation and modification.

#### Differential* NtUGT *gene expression under low-temperature and drought stress

**Fig. 4:**
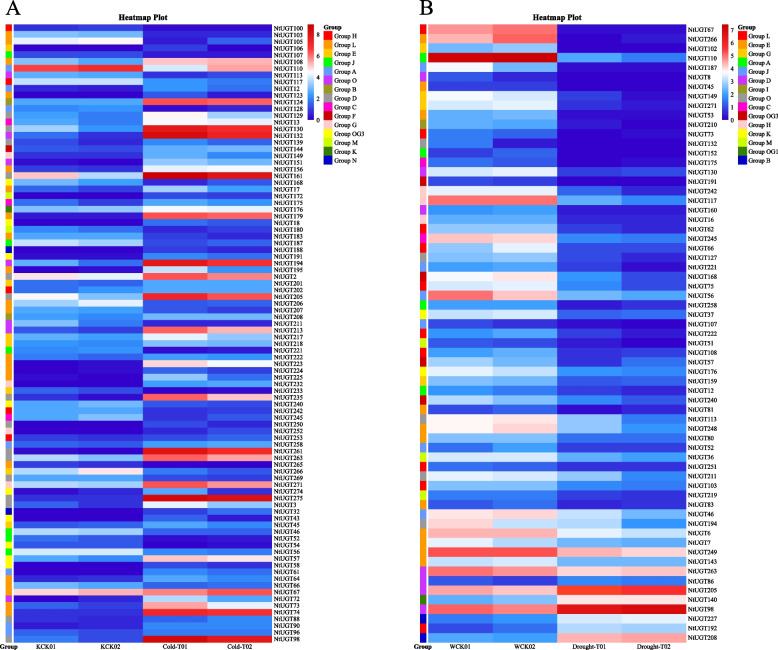
Significantly differentially expressed *NtUGT* genes in response to cold and drought stress. **A** *NtUGT* genes differentially expressed in response to cold stress. **B** *NtUGT* genes differentially expressed in response to drought stress. *NtUGT* genes were classified as differentially expressed using threshold values of |log_2_(FoldChange)|≥ 1 and *p* ≤ 0.05

Previously published tobacco abiotic stress datasets were analyzed. Using thresholds of |log_2_(FoldChange)|≥ 1 and *p* ≤ 0.05, 92 *NtUGT*s were classified as differentially expressed genes (DEGs) under low-temperature stress.28 *NtUGT* genes were down-regulated and 64 *NtUGT* genes were up-regulated in response to low-temperature stress. Among them, *NtUGT46*, *NtUGT54*, *NtUGT56*, *NtUGT103*, *NtUGT107*, *NtUGT113*, *NtUGT117*, *NtUGT183*, *NtUGT242*, *NtUGT265*, and *NtUGT269* were significantly down-regulated; *NtUGT90*, *NtUGT108*, *NtUGT124*, *NtUGT144*, *NtUGT156*, *NtUGT179*, and *NtUGT258* were significantly up-regulated; *NtUGT18*, *NtUGT43*, *NtUGT123*, *NtUGT188*, *NtUGT232* and *NtUGT232* were specially induced expressed under cold stress (Fig. 4A). The above results indicated that the significant different expressed *NtUGT* genes might play roles in regulating tobacco resistance to low temperature stress. Under drought stress, 65 *NtUGT*s were identified as DEGs. Interestingly, under drought stress, most *NtUGT*s were down-regulated, and 22 *NtUGT* genes were significantly down-regulated; only *NtUGT208*, *NtUGT192*, *NtUGT202*, *NtUGT98*, and *NtUGT131* were up-regulated, and only *NtUGT98* was significantly up-regulated (Fig. 4B). There were 38 *NtUGT* genes that were significantly differentially expressed in response to both cold and drought stress. Among them, *NtUGT98, NtUGT205*, and *NtUGT208* were up-regulated under both conditions, 17 *NtUGT* genes were down-regulated under stress conditions compared with the control, and the remaining 18 *NtUGT* genes were up-regulated under cold stress while down regulated under drought stress (Fig. 4).

#### Differential* NtUGT *gene expression between white and pink flowers

**Fig. 5:**
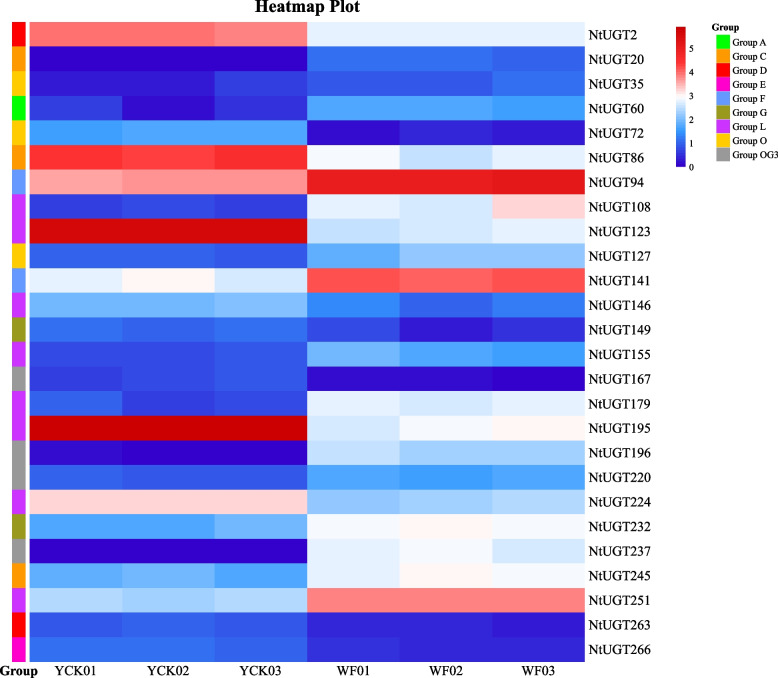
Significantly differentially expressed NtUGTs involved in flavonoid biosynthesis. NtUGTs were classified as differentially expressed using threshold values of |log_2_(FoldChange)|≥ 1 and *p* ≤ 0.05

Using thresholds of |log_2_(FoldChange)|≥ 1 and *p* ≤ 0.05, 26 *NtUGT*s were identified as DEGs between flower tissues of two different colors (Fig. 5). Compared with white flowers (WF), a total of 12 *NtUGT* genes were significantly up-regulated in pink flowers (YCK), including *NtUGT195*, *NtUGT123*, *NtUGT72*, *NtUGT86*, *NtUGT146*, *NtUGT149*, *NtUGT167*, *NtUGT224*, *NtUGT263*, *NtUGT266*, and *NtUGT2*. In contrast, 14 *NtUGT* genes were significantly up-regulated in WF compared to YCK, including *NtUGT20*, *NtUGT60*, *NtUGT94*, *NtUGT108*, *NtUGT127*, *NtUGT141*, *NtUGT155*, *NtUGT179*, *NtUGT196*, *NtUGT220*, *NtUGT232*, *NtUGT245*, *NtUGT237*, and *NtUGT251*.

12 *NtUGT* genes were differentially expressed in both cold-treated plants and between differently-colored flowers. Among them, *NtUGT2*, *NtUGT72*, *NtUGT123*, *NtUGT149*, *NtUGT195*, *NtUGT224*, and *NtUGT263* were up-regulated both in cold stress and in YCK, whereas *NtUGT108*, *NtUGT179*, and *NtUGT232* were up-regulated both in cold stress in WF. 7 *NtUGT* genes were differentially expressed in both under drought stress and between differently-colored flowers. *NtUGT149*, *NtUGT263*, and *NtUGT266* were down-regulated both in drought stress and in WF, whereas *NtUGT108*, *NtUGT127*, *NtUGT245*, and *NtUGT251* were down-regulated both in drought stress in YCK. Five identical *NtUGT* genes (*NtUGT108*, *NtUGT149*, *NtUGT245*, *NtUGT263*, and *NtUGT266*) were differentially expressed in low-temperature stress, drought stress, and differently-colored flowers.

### qRT-PCR validation of DEGs

qRT-PCR analysis was used to confirm the expression patterns of nine randomly-selected *NtUGT*s identified as DEGs based on the RNA-Seq data. The qRT-PCR results showed that *NtUGT88*, *NtUGT108*, *NtUGT123*, *NtUGT179*, *NtUGT195*, and *NtUGT140* were significantly up-regulated under cold stress compared to control plants, which almost consist with the expression of *NtUGT88*, *NtUGT108*, *NtUGT123*, *NtUGT179*, and *NtUGT195* that were detected up-regulated in RNA-Seq data. Under drought stress, *NtUGT86*, *NtUGT227*, and *NtUGT140* were detected up-regulated, whereas *NtUGT108* was down-regulated both in qRT-PCR analysis and in RNA-Seq analysis (Fig. 4 and Figure S6A). Meanwhile, *NtUGT108*, *NtUGT179*, and *NtUGT141* were up-regulated and *NtUGT86*, *NtUGT123*, and *NtUGT195* were down-regulated in WF both in qRT-PCR analysis and in RNA-Seq analysis. (Fig. 5 and Figure S6B). The expression trends of these nine genes were thus consistent with the RNA-Seq data, validating the utility of the transcriptome data.

### Substrate specificity of seven recombinant NtUGT proteins

**Fig. 6:**
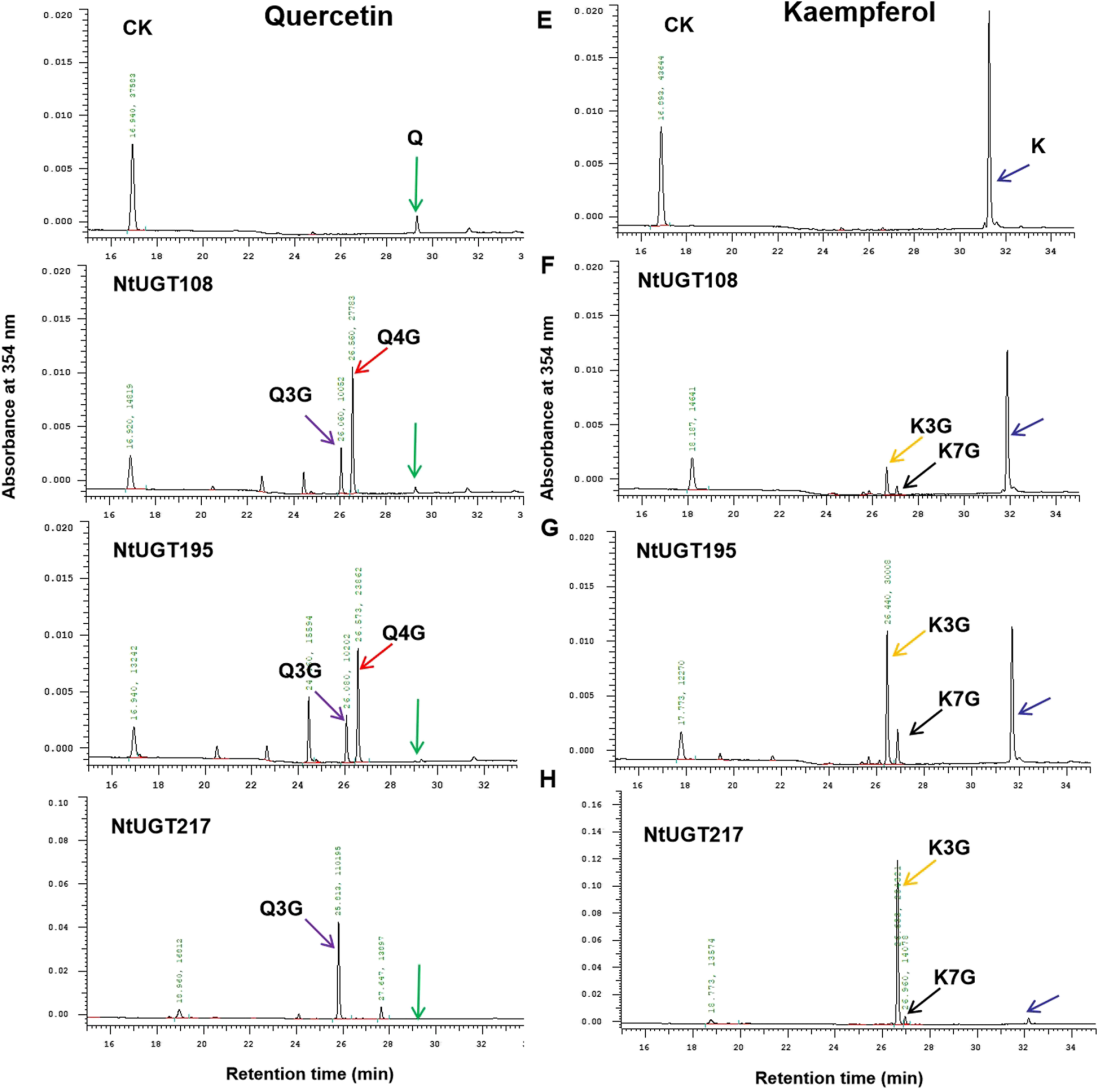
Analysis of the enzymatic reaction products of three recombinant NtUGT proteins. **A**–**D** Representative HPLC chromatograms of the products formed by action of the following NtUGT proteins on quercetin: (**A**) control (no enzyme); (**B**) NtUGT108; (**C**) NtUGT195; and (**D**) NtUGT217. **E**–**H** Representative HPLC chromatograms of the products formed by action of NtUGT proteins on kaempferol: (**E**) control (no enzyme); (**F**) NtUGT108; (**G**) NtUGT195; and (**H**) NtUGT217. Q, quercetin (green arrow); Q3G, quercetin 3-O-glucoside (purple arrow); Q4G, quercetin 4-O-glucoside (red arrow); K, kaempferol (blue arrow); K3G, kaempferol 3-O-glucoside (orange arrow); Q7G, kaempferol 7-O-glucoside (black arrow)

To investigate the enzymatic activity of NtUGT proteins predicted to function in flavonoid biosynthesis, seven candidate *NtUGT* genes with differential expression between white and pink flowers were selected for further enzymatic analysis. The seven candidate *NtUGT* genes were cloned and expressed in *E. coli*. The recombinant proteins were then evaluated in enzymatic assays using four flavonoid aglycones as sugar acceptors and UDP-glucose as the initial sugar donors. The enzymatic products of the seven NtUGTs were identified via HPLC. We found that all seven NtUGTs had activity on myricetin, and six NtUGTs (NtUGT108, NtUGT123, NtUGT141, NtUGT155, NtUGT179, and NtUGT195) showed activity on cyanidin. Three NtUGTs (NtUGT108, NtUGT195, and NtUGT217) also showed activity on flavonol aglycones, namely kaempferol and quercetin (Table 3). Based on the authentic reference standards, the enzymatic products of NtUGT108, NtUGT195, and NtUGT217 had flavonol monoglucosides at different OH groups (3, 4, and 7) (Fig. 6). These were identified as kaempferol 3-O-glucoside (K3G), quercetin 3-O-glucoside (Q3G), quercetin 4-O-glucoside (Q4G), and kaempferol 7-O-glucoside (K7G). NtUGT217 was identified as having much higher enzymatic activity on flavonols, ~ tenfold higher than NtUGT108 and NtUGT195 had. The products of quercetin catalyzed by NtUGT108 and NtUGT195 were identified as mainly Q4G with some Q3G, whereas NtUGT217 produced primarily Q3G (Fig. 6). NtUGT108, NtUGT195, and NtUGT217 primarily catalyzed formation of K3G (and small amounts of K7G) from kaempferol. These results strongly suggested that NtUGT108, NtUGT195, and NtUGT217 acted on flavonol, with strict regio-specificity at the 3-, 4-, and 7-OH positions, respectively.

**Table 3:** Analysis of the enzymatic reaction products of six recombinant NtUGT proteins

**Protein name**	**Substrate name**			
	Kaempferol	Quercetin	Myricetin	Cyanidin
NtUGT108	+	+ +	+ +	+
NtUGT123	-	-	+	+ +
NtUGT141	-	-	+ +	+
NtUGT155	-	-	+ +	+ +
NtUGT179	-	-	+	+
NtUGT195	+ +	+ +	+ +	+
NtUGT217	+ +	+ +	+	-

+  + indicates that the enzymatic product was detected strongly; + indicates that the enzymatic product was detected weakly; – indicates that the enzymatic product was not detectable under the given conditions

In order to elucidate the enzymatic properties of three flavonol aglycone related recombinant NtUGT proteins (NtUGT108, NtUGT195, and NtUGT217), their enzyme kinetic parameters were further determined with UDP-glucose as substrate and compared with each other. The results showed that three NtUGTs had different *Km* values for quercetin 3-O-glucoside (Q3G), quercetin 4-O-glucoside (Q4G), kaempferol 3-O-glucoside (K3G) and kaempferol 7-O-glucoside (K7G) (Table 4). For quercetin as substrate, NtUGT217 got the lowest *Km* value (1.19 × 10–13 mM) and the high *Kcat/Km* value (541,044,022.1 s-1 M-1) for produce quercetin 3-O-glucoside (Q3G), indicating NtUGT217 were the most production efficient quercetin 3-O-glucoside than other than NtUGT108 and NtUGT195. While NtUGT108 showed higher activity toward quercetin 4-O-glucoside (Q4G) than Q3G, NtUGT195 showed the low activity toward Q4G and Q3G. For Kaempferol, three NtUGT proteins showed higher activity toward kaempferol 3-O-glucoside (K3G) than kaempferol 7-O-glucoside (K7G).

**Table 4:** Enzymatic kinetic parameters of NtUGT195, NtUGT108, NtUGT217 proteins with flavonoids as substrates and UDP-glucose as sugar donor

**UGTs**	**Substrates**	**Products**	**Vmax (nmol s **^**−1**^**)**	**Km (mM)**	**Kcat (s **^**−1**^**)**	**Kcat/Km (s**^**−1**^** M**^**−1**^
NtUGT195	Quercetin	Q-3-G	1.74 × 10^–1^	4.38 × 10^–1^	3.01 × 10^–4^	6.86 × 10^–4^
		Q-4-G	1.05 × 10^–1^	5.51 × 10^–3^	1.81 × 10^–4^	3.28 × 10^–2^
	Kaempferol	K-3-G	6.08 × 10^–2^	3.94 × 10^–3^	5.26 × 10^–5^	1.34 × 10^–2^
		K-7-G	6.25 × 10^–1^	5.38 × 10^–3^	5.41 × 10^–4^	1.00 × 10^–2^
NtUGT108	Quercetin	Q-3-G	4.47 × 10^–2^	1.28 × 10^–1^	7.74 × 10^–5^	6.06 × 10^–4^
		Q-4-G	1.16 × 10^–1^	3.38 × 10^–12^	2.01 × 10^–4^	59,482,345.18
	Kaempferol	K-3-G	4.25 × 10^–2^	5.40 × 10^–3^	3.68 × 10^–5^	6.81 × 10^–3^
NtUGT217	Quercetin	Q-3-G	5.83 × 10^–2^	1.19 × 10^–13^	1.04 × 10^–4^	541,044,022.1
		Q-4-G	76.48	4.38 × 10^–1^	1.36 × 10^–1^	3.10 × 10^–1^
	Kaempferol	K-3-G	24.42	6.56 × 10^–2^	2.17 × 10^–2^	3.30 × 10^–1^
		K-7-G	2.14	2.04 × 10^–1^	1.90 × 10^–3^	9.31 × 10^–3^

### *NtUGT217 *overexpression in* N. tabacum*

**Fig. 7:**
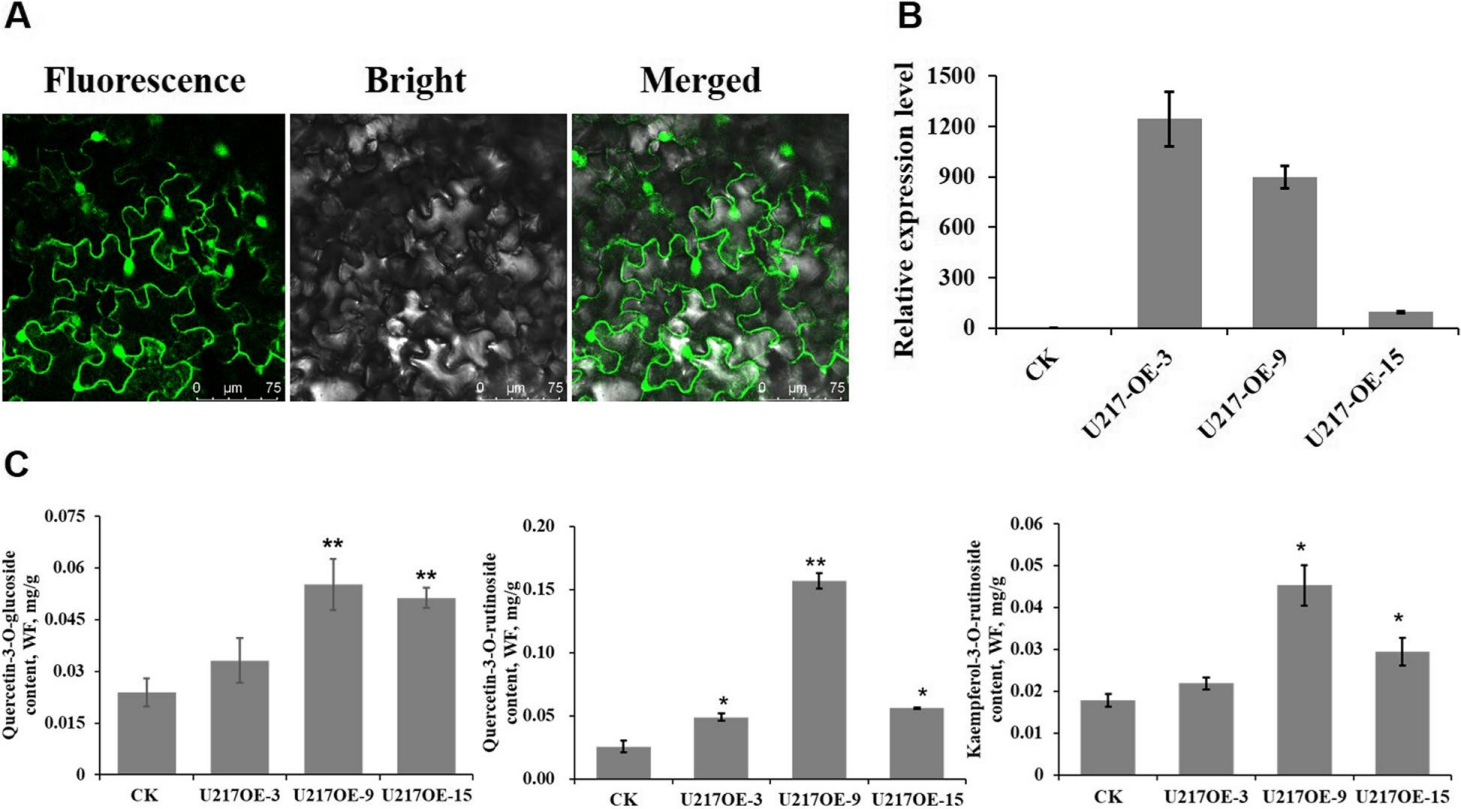
Flavonol glucoside contents in transgenic tobacco overexpressing *NtUGT217*. **A** Subcellular localization analysis of *NtUGT217* gene. Left, confocal micrograph showing green fluorescent protein (GFP) fluorescence; middle, the corresponding differential interference contrast (bright field) image; right, merged fluorescent and bright field image. Scale bars = 20 μm. **B** Expression levels of *NtUGT217* in transgenic tobacco lines overexpressing *NtUGT217*. **C** Levels of quercetin-3-O-glucoside, quercetin-3-O-rutinoside and kaempferol-3-O-rutinoside were dramatically increased in *NtUGT217*-overexpression lines

To evaluate the functions of *NtUGT* genes in flavonol biosynthesis in vivo, we overexpressed *NtUGT217* in tobacco via *Agrobacterium*-mediated transformation. Three transgenic lines with high transcript levels (*U217OE-3*, *U217OE-9*, and *U217OE-15*) were detected by qRT-PCR (Fig. 7B) and analyzed in detail. The flavonols in tobacco leaves were quercetin-3-O-rutinoside and kaempferol-3-O-rutinoside. We detected increased levels of both of those two compounds and quercetin-3-O-glucoside in all three transgenic *NtUGT217*-overexpression tobacco lines (Fig. 7C). Subcellular localization analysis showed that *NtUGT217* was localized to both the nucleus and cytoplasm (Fig. 7A). This was consistent with the localization of nearly all structural proteins involved in flavonoid biosynthesis, such as *CHS*, *CHI*, and *FLS*. Cellular co-localization of those proteins provided potential possibility of protein–protein interactions and functioning in flavonoid biosynthesis. Above all, the results suggested that *NtUGT217* may have important roles in flavonol glycosylation.

## Discussion

Multi-gene UGT families have been identified in several plant species. However, to our knowledge, no further information has been reported about the *UGT* gene family in *N. tabacum*. In this study, we sought to identify and determine putative functions of all *UGT* genes in tobacco. In total, we identified 276 putative *NtUGT* genes (Fig. 1), which encoded proteins of a large range of amino acids lengths. The intron numbers of the 276 *NtUGT* family members varied from 0 to 16, and the gene structure was shown to be complex. Of the 276 putative *NtUGT* genes, 38% lacked introns, which was lower than the rate of 58%, 55%, and 60% of genes lacking introns in *Arabidopsis*, flax, and maize, respectively [31, 32]. Phylogenetic analysis revealed that the 276 NtUGTs were distributed among 18 subgroups, namely 14 conserved subgroups (subgroups A–N), which were present in all species [5]; one newly discovered subgroup (subgroup O), which was found in some higher plant species such as apple, peach, poplar, and maize [33]; and three out-group subgroups (OG1-OG3), which were not subject to known subgroups. Subgroup O contained the most *NtUGT* genes (40), accounting for ~ 14% of all *NtUGT* genes identified in tobacco. Expansion of this subgroup in tobacco indicated that they have performed vital functions associated with tobacco evolution, and their absence in *Arabidopsis* indicates they were lost during the evolution of *Arabidopsis* or that the family expanded after separation from the last common ancestor of tobacco and *Arabidopsis*. The new subgroup OG3 contained the third highest number of *NtUGT* genes (33), indicating their indispensable functions specific to speciation (i.e., formation, adaption, and development) of *N. tabacum*. Expansions of subgroups A, C, D, E, G, I, J, K, L, M, and N in tobacco compared to *Arabidopsis* indicated that multiple functions were associated with these subgroups of UGTs, and they had broad substrate specificity. Subgroups B and F were not found to be expanded, suggesting that they had conserved substrate specificity (Fig. 1). Surprisingly, subgroup H showed a decreased number of NtUGTs in tobacco compared to *Arabidopsis*, with a shrinking of the gene family, which indicated that these *NtUGTs* were partially lost during evolution, which may have limited their functions in tobacco (Fig. 1). Formation, expansion, conservation, and reduction of *NtUGT* genes in each phylogenetic subgroup reflect the evolutionary challenges that plants must overcome to survive.

In multiple plant species, UGTs have been reported to respond to various abiotic stress conditions, including low temperature, drought, and high salt [11, 34]. In this study, gene expression was analyzed to better understand the roles of tobacco *NtUGT* genes in flavonoid biosynthesis and in drought and cold stress resistance. The promoter sequences of *NtUGTs* were shown to contain many cis-elements related to responses to abiotic stressors (such as light, temperature, and drought) and to hormones and flavonoid biosynthesis, suggesting that *NtUGTs* may be involved in responses to a range of stressors. An increasing number of *UGT* genes have been shown to function in cold resistance in multiple plants, including *AtUGT79B2* and *AtUGT79B3* [35], *TaUGT91Q2* [28], *EjUGT92* and *EjUGT88* [36], and *MdUGT83L3* [37]. Under cold stress, we identified 26 significantly up-regulated *NtUGT* genes and eight that were significantly down-regulated. Cold stress response elements such as ABRE, DRF, W-box, ARE, G-box were present in the promoter fragments of those significant different expressed *NtUGT* genes under cold stress, which suggested their potential roles in cold resistance.

*UGT* genes have also been shown to function in drought resistance, include *OsUGT85E1* [38], *AhUGT71K1* and *AhUGT73B4* [39], *UGT003* and *UGT024* in alfalfa (*Medicago sativa*) [40], *AtUGT74E2* [41], *AtUGT71C5* [42], *AtUGT76E11* [43], and *ZmUGT2* [44]. Interestingly, of the 30 *NtUGT* genes significantly differentially expressed under drought stress, the majority (25) were significantly down-regulated; and five *NtUGT* genes were significantly up-regulated (Fig. 4B). The expression patterns of *NtUGT*s were different compared to *MsUGT*s, most of which were significantly up-regulated under drought stress [40]. RNA interference of *UGT75C1* in *Solanum lycopersicum* reportedly enhances the ability of transgenic tomatoes to resist drought stress [45]. Meanwhile, drought stress response elements such as ABRE and W-box were almost present in the promoter fragments of those significant different expressed *NtUGT* genes, which suggested their potential roles in drought resistance. In general, combined with the drought response element and the expression patterns of *NtUGT*s under drought stress, suggested that most of the different expressed *NtUGT*s might have repressive roles in drought resistance.

Several genes involved in the general flavonoid biosynthesis pathway have been characterized in tobacco. However, enzymes encoded by *UGT* genes identified as catalyzing the final glycosylation steps in flavonoid biosynthesis were rare, and remained to be fully characterized. Members of the UGT71/72/73/75/78/79/83/91/94 families have been identified in flavonoid biosynthesis pathways in a range of plants [18, 20, 46-49]. In this study, we investigated the involvement of *NtUGT* genes in flavonoid biosynthesis in two ways. First, we analyzed RNA-Seq data from pink and white tobacco flowers, from which we identified 22 differentially expressed *NtUGT*s. Among them, except for *NtUGT* genes included in *UGT71* (*NtUGT217*), *UGT73* (*NtUGT2*), *UGT74* (*NtUGT123*, *NtUGT195*, and *NtUGT224*), *UGT91* (*NtUGT60*) family, *NtUGT* genes in the *UGT89* family (*NtUGT124*, *NtUGT127*, and *NtUGT179*) and in the *UGT90* family (*NtUGT20*, *NtUGT86*, and *NtUGT245*) were identified for first time as differentially expressed in pink or white flowers. This indicated that they functioned in flavonoid biosynthesis. Second, we performed PPI network analysis, using NtUGTs as bait proteins to identify interacting proteins. NtUGT60, NtUGT94, NtUGT162, NtUGT170, and NtUGT192 were found to interact with some key genes involved in flavonoid biosynthesis, including *CHS*, *CHI*, *LDX*, and three *P450* genes. This suggested that those *NtUGT* genes may function in flavonoid biosynthesis.

Some *NtUGT* genes were detected as differentially expressed in multiple conditions, namely in response to both cold and drought; in response to drought and between differently-colored flowers; or in response to cold and between differently-colored flowers. Nine *NtUGT* genes were differentially expressed in response to both cold and drought treatments. Two *UGT86A1* genes (*NtUGT56* and *NtUGT187*), *NtUGT266*, and *NtUGT206* were significantly down-regulated whereas *NtUGT98* was significantly up-regulated under both conditions. Moreover, all of these five significant different genes contains ABRE motif in their promoter fragment, which a key cis-element response to cold and drought stress. These results suggested that *NtUGT56*, *NtUGT187*, *NtUGT206*, *NtUGT266* and *NtUGT98* may have an important function in cold and drought resistance. Two *NtUGT* genes were differentially expressed in response to drought and between differently-colored flowers. One ZOG gene (*NtUGT127*) was down-regulated both in drought and in white flowers. Another *UGT90A1* (*NtUGT245*) was down-regulated both in drought and in pink flowers. These results suggested that *NtUGT127* and *NtUGT245* may respond to drought stress through modulation of flavonoid contents. Six *NtUGT* genes were differentially expressed both in cold conditions and in different flower colors; all six were up-regulated under cold stress, but three (*NtUGT72*, *NtUGT179*, and *NtUGT195*) were up-regulated in pink flowers whereas the other three (*NtUGT108*, *NtUGT124*, and *NtUGT232*) were up-regulated in white flowers. These results were similar to those observed in other plants. *AtUGT76E11* increases flavonoid accumulation, which enhances drought stress tolerance [49]. *AtUGT79B2* and *AtUGT79B3* enhance tolerance to low temperature stress by increasing anthocyanin accumulation [34]. *MdUGT83L3* increases anthocyanin accumulation in callus tissues and enhances reactive oxygen species (ROS) clearing in response to salt or cold stress exposure [36]. *CsUGT91Q2* can regulate the accumulation of flavonols and scavenge ROS in response to cold treatment [28]. In consideration of the correspondence between flavonoid and stress resistance, *NtUGT72*, *NtUGT179*, and *NtUGT195* may play roles in flavonoid-related cold stress resistance, further investigation of their function in flavonoid biosynthesis and cold resistance should be carried out.

To validate the functions of *NtUGT* genes predicted to be involved in flavonoid biosynthesis based on the RNA-Seq data, enzymatic analyses were performed. In vitro enzymatic assays validated that seven NtUGT proteins showed activity toward various flavonoid substrates, and they were exist in group L (NtUGT108, NtUGT123, NtUGT155, NtUGT179, and NtUGT195), F (NtUGT217) and F (NtUGT141). The enzymatic analysis results consistent with the previous report that UGT proteins enriched in subgroups A, F, L and E were related to flavonoid synthesis and polyphenolic metabolism [4, 5]. All seven selected NtUGTs exhibited activity on myricetin; six NtUGTs (all except NtUGT217) showed activity on cyanidin; and three NtUGTs (NtUGT108, NtUGT195, and NtUGT217) were active on flavonol aglycones, namely kaempferol and quercetin. NtUGT108, NtUGT195, and NtUGT217 catalyzed kaempferol and quercetin more efficiently than myricetin. Compared with NtUGT108 and NtUGT195, NtUGT217 was more efficient on kaempferol and quercetin. NtUGT108 was more efficient on kaempferol than quercetin, whereas the reverse was true of NtUGT195 (Table 4 and Fig. 6). The products of quercetin catalysis by NtUGT108 or NtUGT195 were mainly Q4Gs with some Q3Gs, whereas NtUGT217 primarily produced Q3Gs (Fig. 6). Catalysis of kaempferol by NtUGT108, NtUGT195, and NtUGT217 primarily produced K3Gs and rare K7Gs. With respect to regio-specificity, NtUGT217 belonged to the UGT71 family; these function similarly to UGT71 proteins in strawberry, which prefer 3-hydroxycoumarin as substrates and form 3-glucosides in the flavonol pathway [50]. NtUGT108 and NtUGT195 belonged to the UGT74 family, and were similar to several UGT74 proteins in other plants that produced 3-O-glucosides in the kaempferol pathway [51]; however, they produced Q4G in tobacco, which differed from the quercetin pathways in other plants. Furthermore, *NtUGT217* overexpression was conducted in tobacco to validate its function in flavonoid biosynthesis in vivo. Transgenic lines overexpressing *NtUGT217* showed marked increases in quercetin-3-O-glucoside, quercetin-3-O-rutinoside and kaempferol-3-O-rutinoside accumulation, consistent with the enzymatic activities observed in vitro (Fig. 7).

## Conclusion

In this study, 276 *UGT* genes were identified in *N. tabacum* and were found to form 18 subfamilies. We characterized the chromosomal distribution and gene structure of the genes, predicted their interaction protein using PPI analysis, and then identified *NtUGT* genes which involved in cold and drought tolerance and in flavonoid biosynthesis based on the online RNAseq data. We identified seven NtUGTs that potentially involved in flavonoid glycosylation, and we further investigated the enzymatic products and enzymatic properties of three NtUGT proteins (NtUGT108, NtUGT195, and NtUGT217), which mainly formed 3- or 4- glucosides towards flavonols. Finally, we over-expressed *NtUGT217* in tobacco to validate its function in flavonol biosynthesis. *NtUGT217* overexpression transgenic lines showed increased quercetin-3-O-glucoside, quercetin-3-O-rutinoside and kaempferol-3-O-rutinoside accumulation. This study provides a comprehensive description of *NtUGT* genes in tobacco and explores the key candidate *NtUGT* genes likely to be involved in cold and drought resistance and in flavonoid biosynthesis. This study lays the groundwork for future functional investigations of *NtUGT* genes in *N. tabacum*.

## Methods

### Identification of* NtUGT *genes in* Nicotiana tabacum*

To identify candidate *NtUGT* genes in tobacco, the Hidden Markov Model (HMM) profile corresponding to the UDPGT domain (PF00201) was retrieved from Pfam (http://pfam.xfam.org/). The *N. tabacum* protein database (https://solgenomics.net/ftp/genomes/Nicotiana_tabacum/edwards_et_al_2017/annotation/) was searched using the HMM file and hmmsearch software with an E-value threshold of 1e-5. The conserved PSPG motif sequence (44 aa in length) was also used as a query in BLASTP against the *N. tabacum* protein database. Candidate proteins yielded by the two strategies were screened using SMART (http://smart.emblheidelberg.de) to remove proteins without a complete PSPG motif. All of the identified *NtUGT* genes were classified and named based on chromosomal location (or scaffold location as necessary). MapChart was used to plot images of the physical gene locations in *N. tabacum*. The subcellular localization of each NtUGT protein was predicted with PSORT II Prediction (http//www.genscript.com/psort.html).

Gene ontology (GO) terms and Kyoto Encyclopedia of Genes and Genomes (KEGG) biochemical pathways were analyzed for all putative *NtUGT* genes in tobacco. GO term enrichment among candidate genes was analyzed using the ‘GOseq’ package [52] in R. KOBAS software was used to find statistically enriched KEGG pathways among the candidate genes [53].

### Phylogenetic tree construction

Candidate UGT protein sequences from tobacco and Arabidopsis were aligned with ClustalW in MEGA7 (https://www.megasoftware.net/). 114 Arabidopsis UGT protein sequences were retrieved from CAZy (http://www.cazy.org/GlycosylTransferases.html). Sequences that were too short (< 60 aa) or too divergent from the others were removed from the input file after the initial alignment, then the remaining sequences were re-aligned. Phylogenetic analysis was performed in MEGA7 using the neighbor-joining method with 1000 bootstrap replicates.

### Analysis of gene structure and conserved motifs in* NtUGT *genes

Gene structure data comprised information about exons/introns and untranslated region (UTR) organization. These data were obtained for the predicted *NtUGT* genes from the *N. tabacum* GFF annotation file and illustrated using the Gene Structure Display Server (GSDS) (http://gsds.cbi.pku.edu.cn/) [54]. The conserved motifs of putative UGT proteins were predicted using MEME (http://meme-suite.org/tools/meme) with a maximum of 10 motifs per sequence. *Cis*-acting elements, including promoters, enhancers, regulatory sequences, and inducible elements, were identified using the PlantCARE database (http://bioinformatics.psb.ugent.be/webtools/plantcare/html/), then mapped with the GSDS.

### Ka/Ks ratio analysis of NtUGT genes

The ratios of non-synonymous substitution rates (*Ka*) to synonymous substitution rates (*Ks*) were calculated for *NtUGT* gene pairs in Phylogenetic Analysis by Maximum Likelihood (PAML) [55] to estimate the selection modes. *Ka/Ks* ratios greater than, equal to, and less than 1 were considered to represent positive, neutral, and negative selection, respectively.

### Prediction and analysis of NtUGT protein interactors

The Ortho venn tool (http://www.bioinfogenome.net/OrthoVenn/) was used to identify orthologous UGT gene pairs between tobacco and Arabidopsis and to establish a homologous mapping relationship. Protein interaction networks were then built for NtUGTs based on the orthologous genes between tobacco and Arabidopsis using STRING software (http://string-db.org/cgi). Finally, predicted interaction networks were displayed in Cytoscape (https://cytoscape.org/).

#### Differential expression of *NtUGT* genes involved in flavonoid biosynthesis and in drought and cold stress responses

Transcriptome data were obtained from the National Center for Biotechnology Information (NCBI) Sequence Read Archive (SRA), namely the datasets PRJNA590063 (white and pink tobacco flowers), PRJNA534356 (drought-treated and control tobacco plants) and PRJNA368913 (cold-treated and control tobacco plants). The data were analyzed using DESeq2 software [56] to identify differentially expressed genes. Heatmaps were generated using thresholds of fold-change > 1.5 and p < 0.05.

For quantitative realtime (qRT) PCR analysis, tobacco plants with 4 or 5 fully expanded leaves at 4 weeks were used in cold and drought experiment, leave samples were collected after 24 h-cold-treatment or 48 h-drought treatment, respectively. Corolla of white and pink tobacco flowers were collected, all samples were frozen in liquid nitrogen and stored in -80 ℃ for RNA extraction. qRT-PCR was conducted as described by Chen et al. [57, 58].The Tob103 gene (GenBank accession no. U60495) served as an internal control. Primers for the random selected 9 *NtUGT* genes were listed in Supplementary Table S1. The comparative cycle threshold (2^−ΔΔCT^) method was used to calculate relative expression levels of target genes.

### Expression and purification of NtUGT proteins in Escherichia coli

Primers were designed for 7 candidate *NtUGT* genes (*NtUGT108*, *NtUGT123*, *NtUGT141*, *NtUGT155*, *NtUGT179*, *NtUGT195*, and *NtUGT217*) based on the identified coding region sequences (Supplementary Table S2). The resulting PCR products were purified, then ligated to a pGEX4.0 vector digested with the same restriction enzyme *EcoR I*. The pGEX4.0-NtUGT vectors were sequenced and transformed into *E. coli* strain BL21 competent cells. Recombinant glutathione S-transferase (GST) fusion protein expression was induced with 60 μl of 50 mg/ml isopropyl β-d-1-thiogalactopyranoside (IPTG). After overnight incubation at 16 °C with shaking at 180 rpm, cells were harvested by centrifugation at 4 °C, then stored at -80 °C prior to purification.

Proteins were affinity-purified using a GST protein fusion and purification system. In brief, cell pellets were re-suspended in lysis/wash buffer containing 50 mM HEPES, 150 mM NaCl, 1 mM EDTA, 1 mM PMSF, and 1 mM dithiothreitol (DTT) at pH 7.4. Samples were then sonicated for 30 min with a 3 s interval stop each cycle on ice. Crude protein extracts were loaded into a column packed with GST-binding beads to bind the GST fusion proteins, which were eluted with lysis/wash buffer. The recombinant fusion NtUGT proteins were detected via SDS-PAGE and visualized by staining with Coomassie brilliant blue. Protein concentration was measured using bovine serum albumin (BSA) as the standard.

### Enzymatic assay and product identification

Recombinant NtUGT proteins (40 μg each) were incubated at 30 °C with 100 mM Tris–HCl (pH 7.0), 0.5 mM substrate, and 5 mM UDP-glucose in a final volume of 500 µl. After 30 min, reactions were stopped with the addition of 500 μl methanol, then centrifuged at 2200 × g for 10 min. Samples were analyzed using high-performance liquid chromatography (HPLC).

For kinetic analysis, purified 15 μg each of NtUGT108, NtUGT195 and NtUGT217 were added to reaction mixtures with 100 mM Tris–HCl (pH 7.0) and 5 mM UDP-glucose in a final volume of 500 µl. The concentration of the tested flavonoid substrates ranged from 0–400 µM (0, 10, 50, 100, 150, 200, 250, 300, 350, 400 µM). Reactions were stopped with addition of 500 µl methanol after 30 min incubation at 30 °C. Samples were centrifuged at 2200 xg for 10 min and further analyzed by HPLC. The Kinetic parameters *Vmax* and *Km* were calculated by using GraphPad Prism.

Enzymatic products were separated via HPLC with a linear A:B elution gradient from 95% solvent A (0.2% acetic acid) to 95% solvent B (100% acetonitrile) over a 48 min period with a flow rate of 1 ml min^−1^. Reaction products were monitored using a diode array detector at 345 nm for flavonols (quercetin, kaempferol, and myricetin) and at 275 nm for anthocyanin.

### Subcellular localization of* NtUGT217*

To determine the subcellular localization of pRI101-*NtUGT217*-eGFP, the primer pair NtUGT217-eGFP-F/NtUGT217-eGFP-R (Supplementary Table S3) was used to amplify the NtUGT217 coding region. The PCR product was cloned into the *BamHI*-digested pRI101-eGFP plasmid using the ClonExpress Entry One Step Cloning Kit (Vazyme Biotech, China). *Agrobacterium tumefaciens* strain EHA105 was transformed with the pRI101-*NtUGT217*-eGFP construct; *Nicotiana benthamiana* leaves were infiltrated with transformed bacteria and examined as described by Chen et al. [58].

### *NtUGT217 *overexpression in tobacco

To construct the overexpression vector p1305-*NtUGT217,* the primer pair NtUGT217-p1305-F/NtUGT217-p1305-R (Supplementary Table S3) was used to amplify the *NtUGT217* coding region. The open reading frame (ORF) region of the *NtUGT217* gene driven by the double cauliflower mosaic virus (CaMV) 35S promoter was inserted into the *BamHI*-digested binary vector pCAMBIA1305. *Agrobacterium* strain LBA4404 was transformed with the p1305-*NtUGT217* construct, then tobacco was transformed using the leaf disc method as reported by Horsch et al. [59].

#### Measurement of quercetin-3-O-glucoside, quercetin-3-O-rutinoside and kaempferol-3-O-rutinoside via HPLC

Quercetin-3-O-glucoside, quercetin-3-O-rutinoside and kaempferol-3-O were extracted and measured as described by Chen et al. [58]. Analyses were carried out on three independent biological replicates, for which there were three technical replicates each.

## Supplementary Information

**Additional file1: Table S1.** Primers used for qRT-PCR amplification.

**Additional file 2: Table S2.** Primers used for NtUGT genes amplification.

**Additional file 3: Table S3.** Primers used in vector construction to determine NtUGTs subcellular localization and for NtUGT217 overexpression.

**Additional file 4: Table S4.** Subcellular location of 276 *NtUGTs* in *Nicotiana tabacum.*

**Additional file 5: Table S5.** Gene Ontology (GO) terms and Kyoto Encyclopedia of Genes and Genomes (KEGG) biochemical pathways associated with *NtUGTs*.

**Additional file 6: Table S6.** Intron/exon data for *NtUGT* genes.

**Additional file 7: Figure S1.** The predicted subcellular location distribution of *NtUGT *genes.

**Additional file 8: Figure S2.** GO and KEGG analysis of* NtUGT* genes in *Nicotiana tabacum.* A. GO term enrichment analysis results. B. KEGG pathway enrichment analysis results.

**Additional file 9: Figure S3.** Chromosome distribution of 123 *NtUGT* genes *in Nicotiana tabacum. NtUGT*s were distributed across 24 chromosomes. Green colored bars represent chromosomes; chromosome numbers are given at the top of each bar. Red boxes indicate genes derived from tandem duplication.

**Additional file 10: Figure S4.** Gene structure analysis of *NtUGT* genes in *Nicotiana tabacum*. A.Gene structure and their phylogenetic results of *NtUGT* genes. Green box indicates exons and dark lines indicates introns of *NtUGT* genes*.* B. Conserved motif analysis of NtUGT proteins. Motifs were marked by different colors.

**Additional file 11: Figure S5.** *Cis-elements distribution of NtUGT genes in Nicotiana tabacum*.

**Additional file 12: Figure S6.** Relative expression levels of selected *NtUGT* genes. A. Relative expression levels of selected *NtUGT* genes in response to cold and drought treatments. B. Relative expression levels of selected *NtUGT* genes in white and pink tobacco flowers.

## Data Availability

The datasets used and/or analyzed during the current study available from the corresponding author on reasonable request. Formal identification of plant materials was undertaken by the corresponding author of this paper (Shuai Chen). No voucher specimen of this material has been deposited in a publicly available herbarium.
