# Supplementary material for: Genome-wide analysis of UDP-glycosyltransferases family and identification of UGT genes involved in abiotic stress and flavonol biosynthesis in Nicotiana tabacum
Source: BMC Plant Biol. 2023 Apr 19;23:204. doi: 10.1186/s12870-023-04208-9 (PMC10114341; doi:10.1186/s12870-023-04208-9)
Supplement: Supplementary file 1 — Additional file1: Table S1. Primers used for qRT-PCR amplification. [file 12870_2023_4208_MOESM1_ESM.docx]

**Supplementary Table S1.** Primers used for qRT-PCR amplification.

| Gene | Primers |
| --- | --- |
| qRTNtUGT86-F | GTGTTATCCAATTGCGGGTGA |
| qRTNtUGT86-R | ACCATCCATTCTAACGCCGA |
| qRTNtUGT88-F | GCTTCACAGAAATGTAAAATC |
| qRTNtUGT88-R | AAAAGTTGGTGATGAGTTTCG |
| qRTNtUGT108-F | TCCTCCATGATCTTCTCAAAT |
| qRTNtUGT108-R | AGGCATGAAAAGTGAAGAA |
| qRTNtUGT123-F | CACATCTGGCCCTGTTTGAT |
| qRTNtUGT123-R | TTGGAAATTTCTGAGTTTACA |
| qRTNtUGT140-F | ACCCATTCTAACTTCCGCGA |
| qRTNtUGT140-R | CCTGGCCATATATCCTGCCA |
| qRTNtUGT141-F | TGCTCTATGTCCCGAATGAA |
| qRTNtUGT141-R | TCCGTGTGACATCATTTTCC |
| qRTNtUGT179-F | TTGCATCCAAAAGTCCAGTT |
| qRTNtUGT179-R | GGTCAATGGGTCATTCCCTA |
| qRTNtUGT195-F | TGTTGAAAGCGCCTTTAGAGT |
| qRTNtUGT195-R | CTTGTATTCCATCGGCCACT |
| qRTNtUGT227-F | CAGTGTCGGATCCTTACAAAA |
| qRTNtUGT227-R | GTTGCTCGAACTCTTCAAAAC |
| Tob103actin-F | ATGAGAGAGTGCATATCGAT |
| Tob103actin-R | TTCACTGAAGAAGGTGTTGAA |
