# Supplementary material for: Genome-wide analysis of UDP-glycosyltransferases family and identification of UGT genes involved in abiotic stress and flavonol biosynthesis in Nicotiana tabacum
Source: BMC Plant Biol. 2023 Apr 19;23:204. doi: 10.1186/s12870-023-04208-9 (PMC10114341; doi:10.1186/s12870-023-04208-9)
Supplement: Supplementary file 2 — Additional file 2: Table S2. Primers used for NtUGT genes amplification. [file 12870_2023_4208_MOESM2_ESM.docx]

**Supplementary Table S2.** Primers used for UGT genes amplification

| Gene | Primers |
| --- | --- |
| UGT108-pGEX-F | CCGCGTGGATCCCCGGAATTCATGAGTACTACTCACAAAGCTC |
| UGT108-pGEX-R | CCGCTCGAGTCGACCCGGGTTAAGAAATAGTCATCAAATTGG |
| UGT123-pGEX-F | CCGCGTGGATCCCCGGAATTCATGAAAATGAGTCAATTCTCCA |
| UGT123-pGEX-R | CCGCTCGAGTCGACCCGGGTTATTTCATGTATTTCTTCATC |
| UGT141-pGEX-F | CCGCGTGGATCCCCGGAATTCATGTCCAATTATCATGTTGCTG |
| UGT141-pGEX-R | CCGCTCGAGTCGACCCGGGTCAATTGCTAGAGGTGATAATC |
| UGT155-pGEX-F | CGTGGATCCCCGGAATTCATGGCAAAACTGAAAGATAATTGTC |
| UGT155-pGEX-R | CCGCTCGAGTCGACCCGGGTTATGCATGGCTATTAAGAACCTC |
| UGT179-pGEX-F | CCGCGTGGATCCCCGGAATTCATGAGTACTACTCACAAAGCTCA |
| UGT179-pGEX-R | CCGCTCGAGTCGACCCGGGTTAAGAAATAGTCGTCAAATTGG |
| UGT195-pGEX-F | CCGCGTGGATCCCCGGAATTCATGGTAGGCAAAAGAAATGAGC |
| UGT195-pGEX-R | CCGCTCGAGTCGACCCGGGTATTTCTTGTACTTCTTCATCTG |
| UGT217-pGEX-F | CCGCGTGGATCCCCGGAATTCATGAGCAAATTAGAGCTGGTGT |
| UGT217-pGEX-R | CCGCTCGAGTCGACCCGGGCTAGGAATCAAGGATAGTTTCG |
