# Supplementary material for: Genome-wide analysis of UDP-glycosyltransferases family and identification of UGT genes involved in abiotic stress and flavonol biosynthesis in Nicotiana tabacum
Source: BMC Plant Biol. 2023 Apr 19;23:204. doi: 10.1186/s12870-023-04208-9 (PMC10114341; doi:10.1186/s12870-023-04208-9)
Supplement: Supplementary file 3 — Additional file 3: Table S3. Primers used in vector construction to determine NtUGTs subcellular localization and for NtUGT217 overexpression. [file 12870_2023_4208_MOESM3_ESM.docx]

**Supplementary Table S3.** Primers used for subcelluar location of NtUGT217

| Gene | Primers |
| --- | --- |
| UGT217-eGFP-F | CCCGGGGGTACCGGATCCATGAGCAAATTAGAGCTGGTGT |
| UGT217-eGFP-R | ACCATGAATTCGGATCCGGAATCAAGGATAGTTTCG |
| UGT217-p1305-F | TAGGAGCTCGGTACCCGGGGATCCATGAGCAAATTAGAGCTGGTGT |
| UGT217-p1305-R | GCAGGTCGACTCTAGAGGATCCCTAGGAATCAAGGATAGTTTCG |
