# Supplementary figures and images for: Genome-wide analysis of UDP-glycosyltransferases family and identification of UGT genes involved in abiotic stress and flavonol biosynthesis in Nicotiana tabacum
Source: BMC Plant Biol. 2023 Apr 19;23:204. doi: 10.1186/s12870-023-04208-9 (PMC10114341; doi:10.1186/s12870-023-04208-9)

## Slide 1
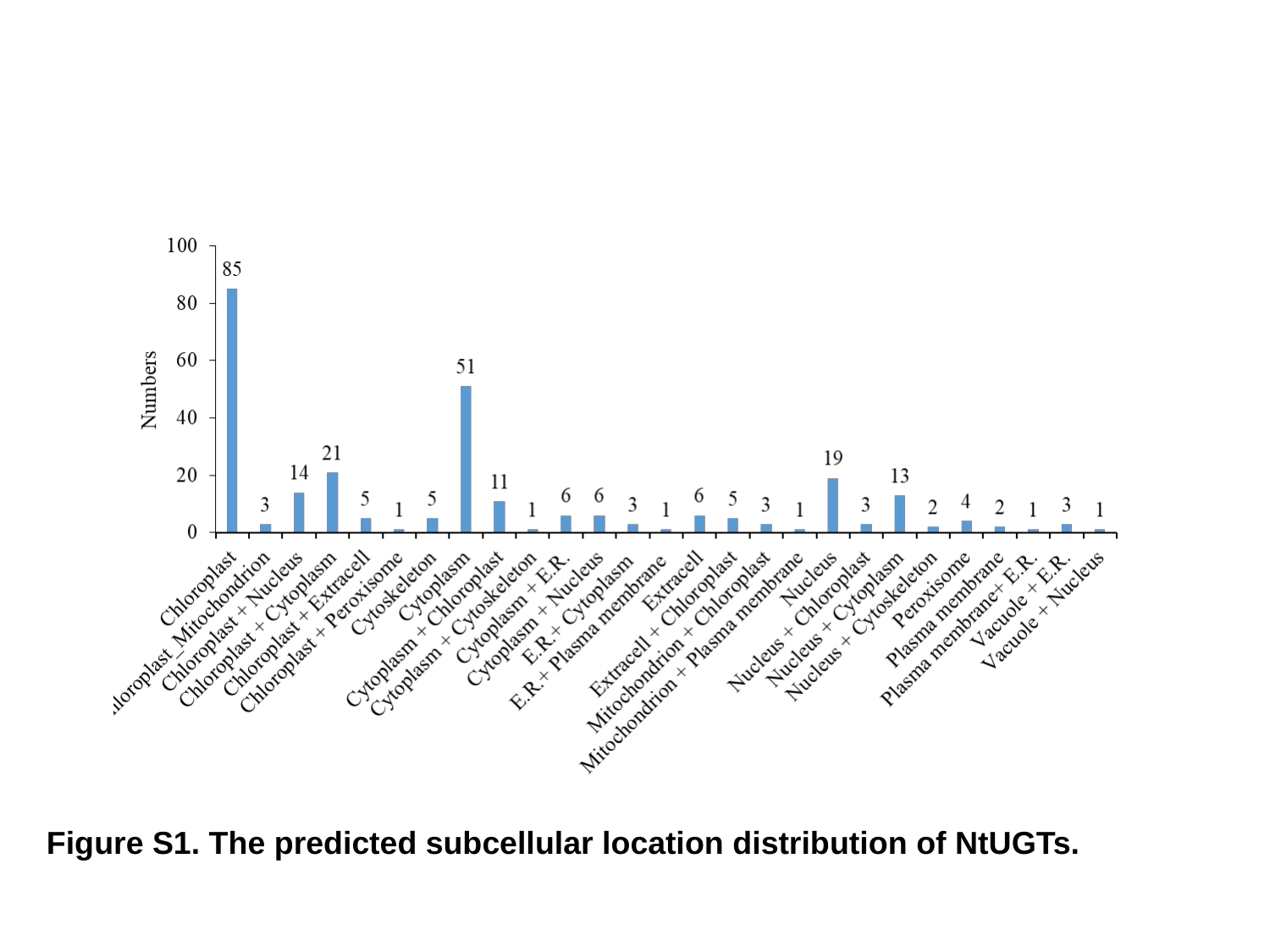

Figure S1. The predicted subcellular location distribution of NtUGTs.

Supplement: Supplementary file 7 — Additional file 7: Figure S1. The predicted subcellular location distribution of NtUGT genes. [file 12870_2023_4208_MOESM7_ESM.ppt]

## Slide 1
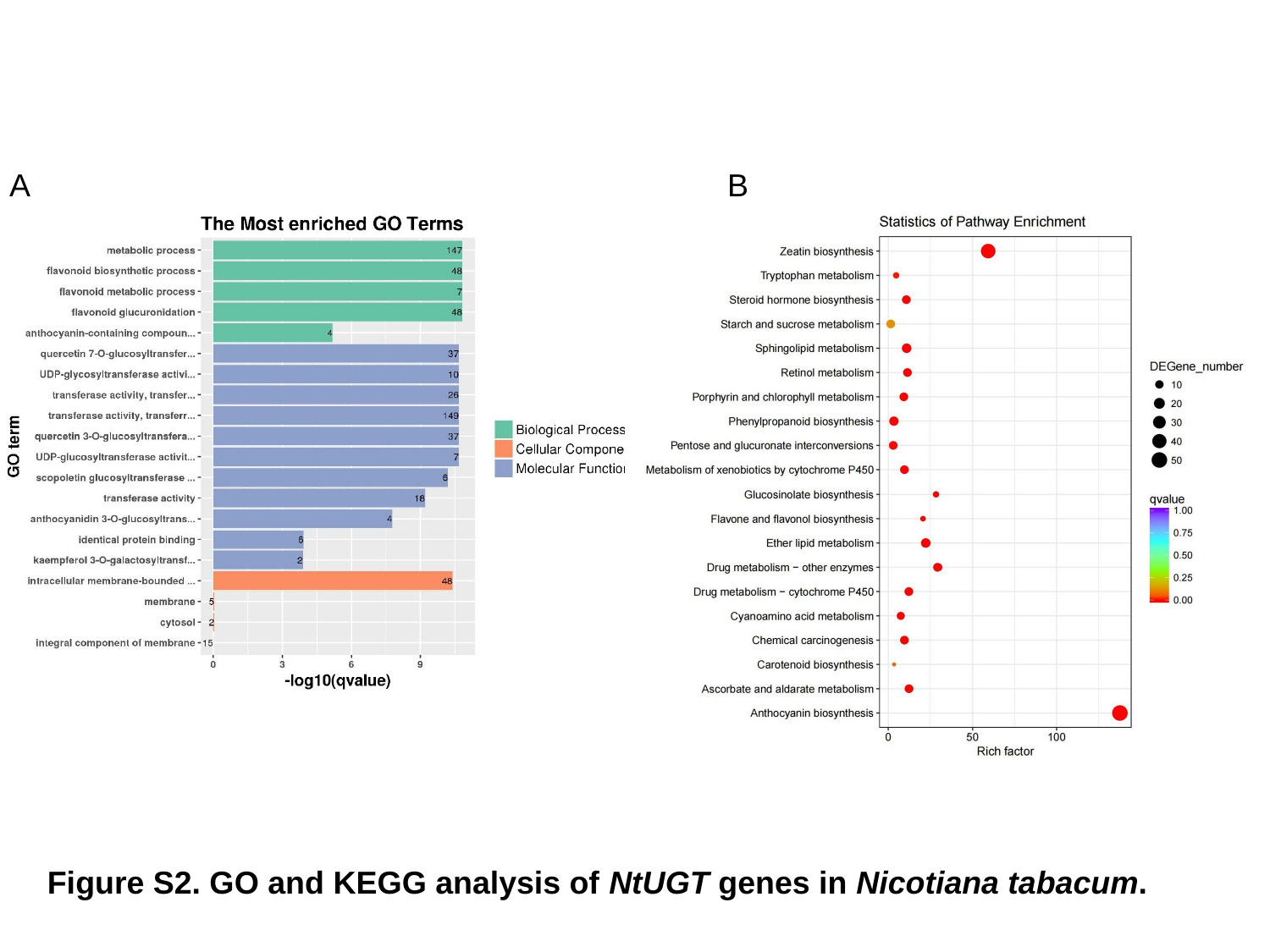

A
B
Figure S2. GO and KEGG analysis of NtUGT genes in Nicotiana tabacum.

Supplement: Supplementary file 8 — Additional file 8: Figure S2. GO and KEGG analysis of NtUGT genes in Nicotiana tabacum. A. GO term enrichment analysis results. B. KEGG pathway enrichment analysis results. [file 12870_2023_4208_MOESM8_ESM.ppt]

## Slide 1
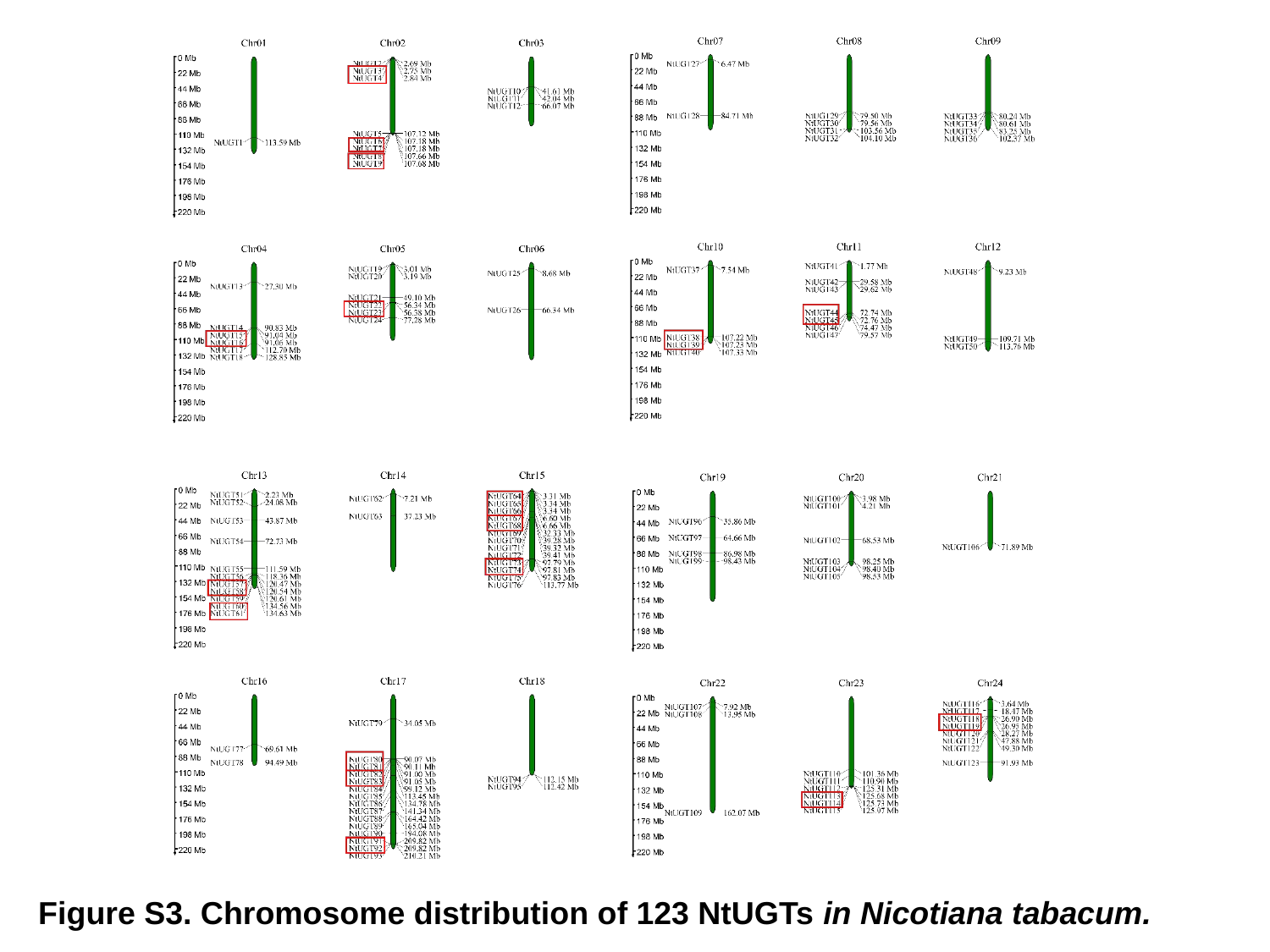

Figure S3. Chromosome distribution of 123 NtUGTs in Nicotiana tabacum.

Supplement: Supplementary file 9 — Additional file 9: Figure S3. Chromosome distribution of 123 NtUGT genes in Nicotiana tabacum. NtUGTs were distributed across 24 chromosomes. Green colored bars represent chromosomes; chromosome numbers are given at the top of each bar. Red boxes indicate genes derived from tandem duplication. [file 12870_2023_4208_MOESM9_ESM.ppt]

A

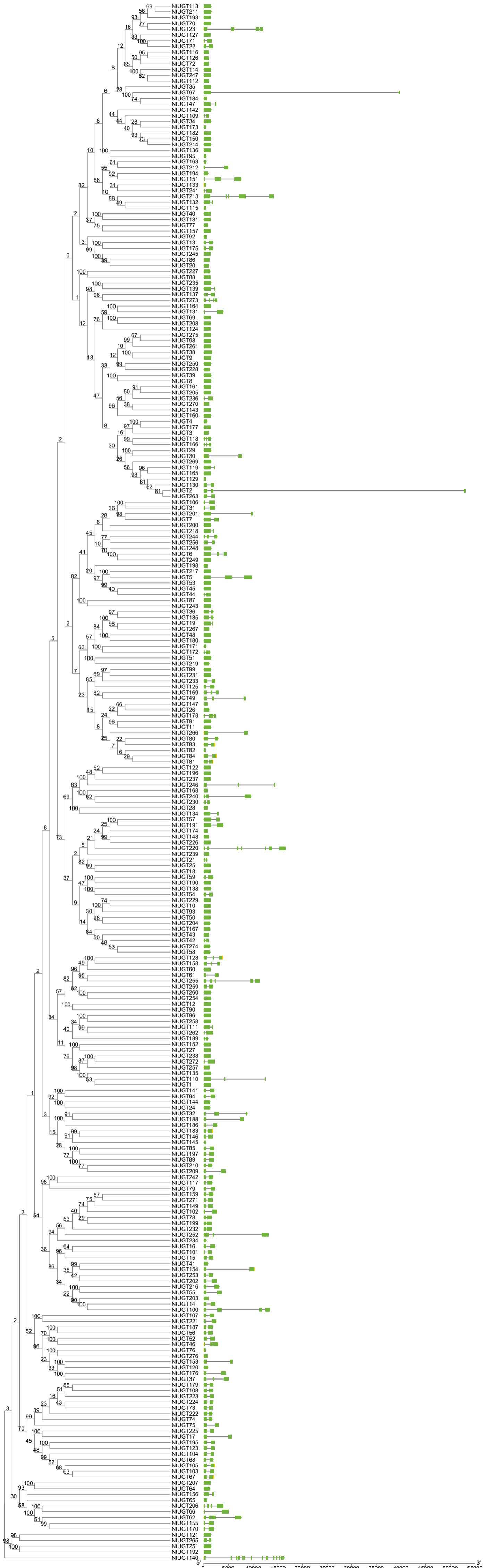

B

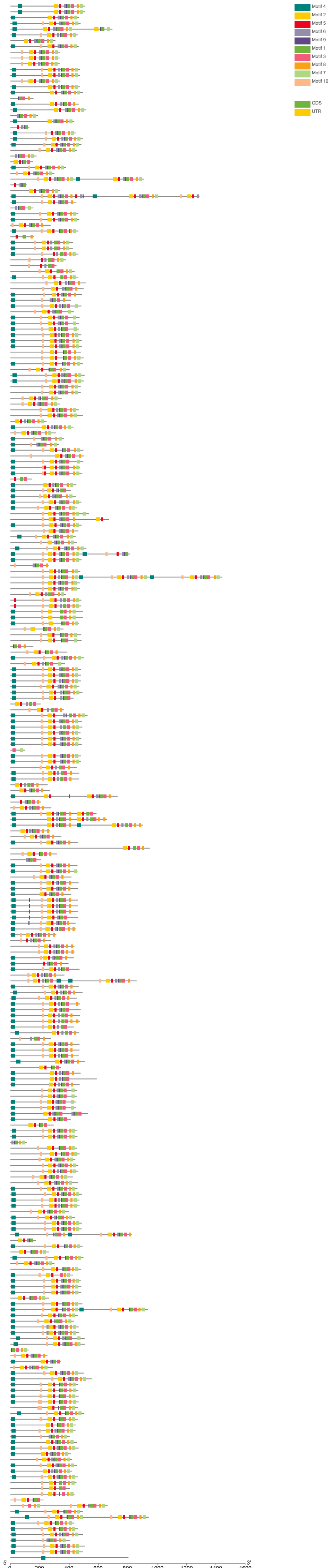

Supplement: Supplementary file 10 — Additional file 10: Figure S4. Gene structure analysis of NtUGT genes in Nicotiana tabacum. A.Gene structure and their phylogenetic results of NtUGT genes. Green box indicates exons and dark lines indicates introns of NtUGT genes. B. Conserved motif analysis of NtUGT proteins. Motifs were marked by different colors. [file 12870_2023_4208_MOESM10_ESM.pdf]

## Slide 1
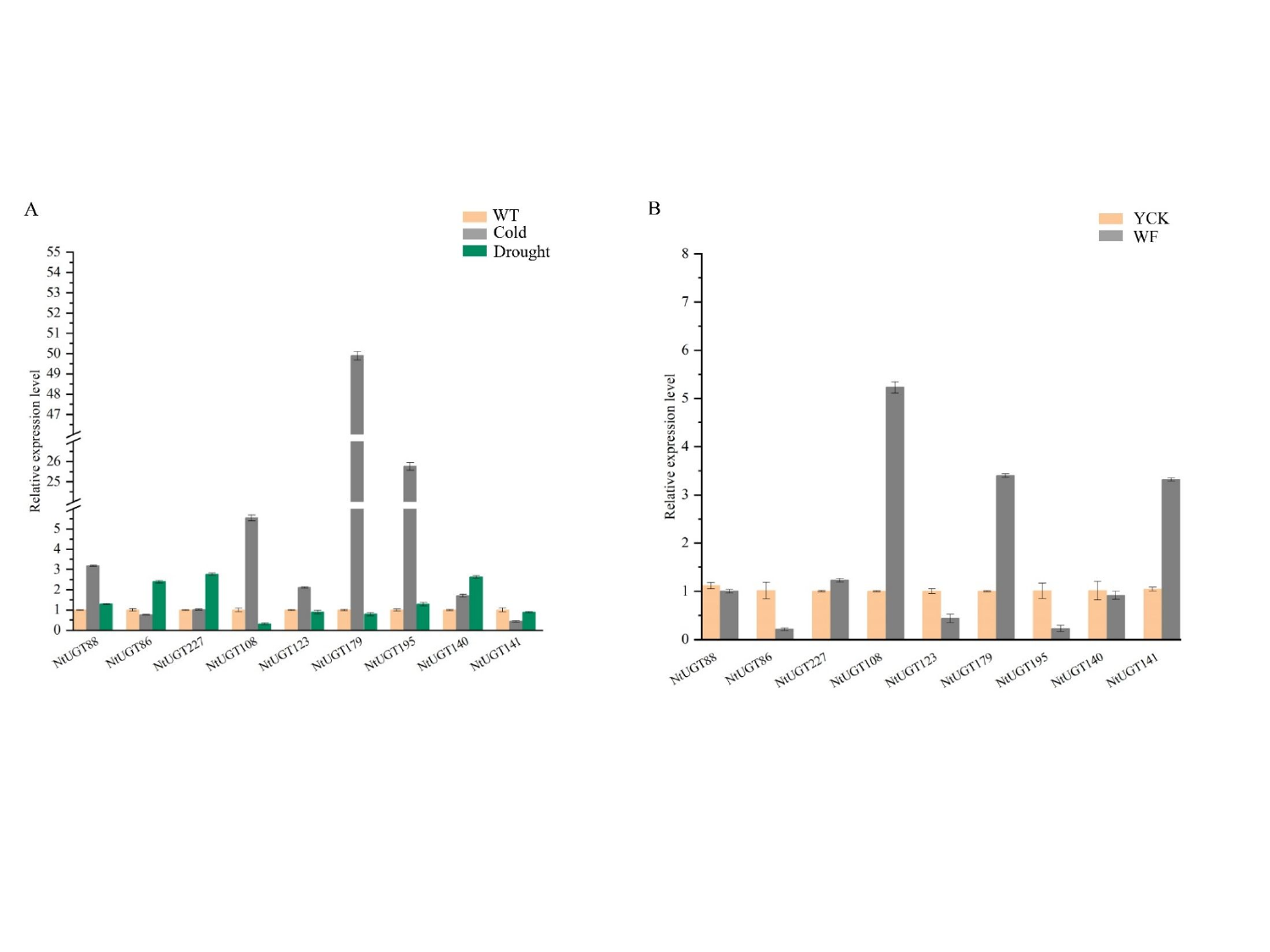

Supplement: Supplementary file 12 — Additional file 12: Figure S6. Relative expression levels of selected NtUGT genes. A. Relative expression levels of selected NtUGT genes in response to cold and drought treatments. B. Relative expression levels of selected NtUGT genes in white and pink tobacco flowers. [file 12870_2023_4208_MOESM12_ESM.ppt]
