# Supplementary material for: Genome-wide analysis of UDP-glycosyltransferases family and identification of UGT genes involved in abiotic stress and flavonol biosynthesis in Nicotiana tabacum
Source: BMC Plant Biol. 2023 Apr 19;23:204. doi: 10.1186/s12870-023-04208-9 (PMC10114341; doi:10.1186/s12870-023-04208-9)
Supplement: Supplementary file 11 — Additional file 11: Figure S5. Cis-elements distribution of NtUGT genes in Nicotiana tabacum. [file 12870_2023_4208_MOESM11_ESM.pdf]

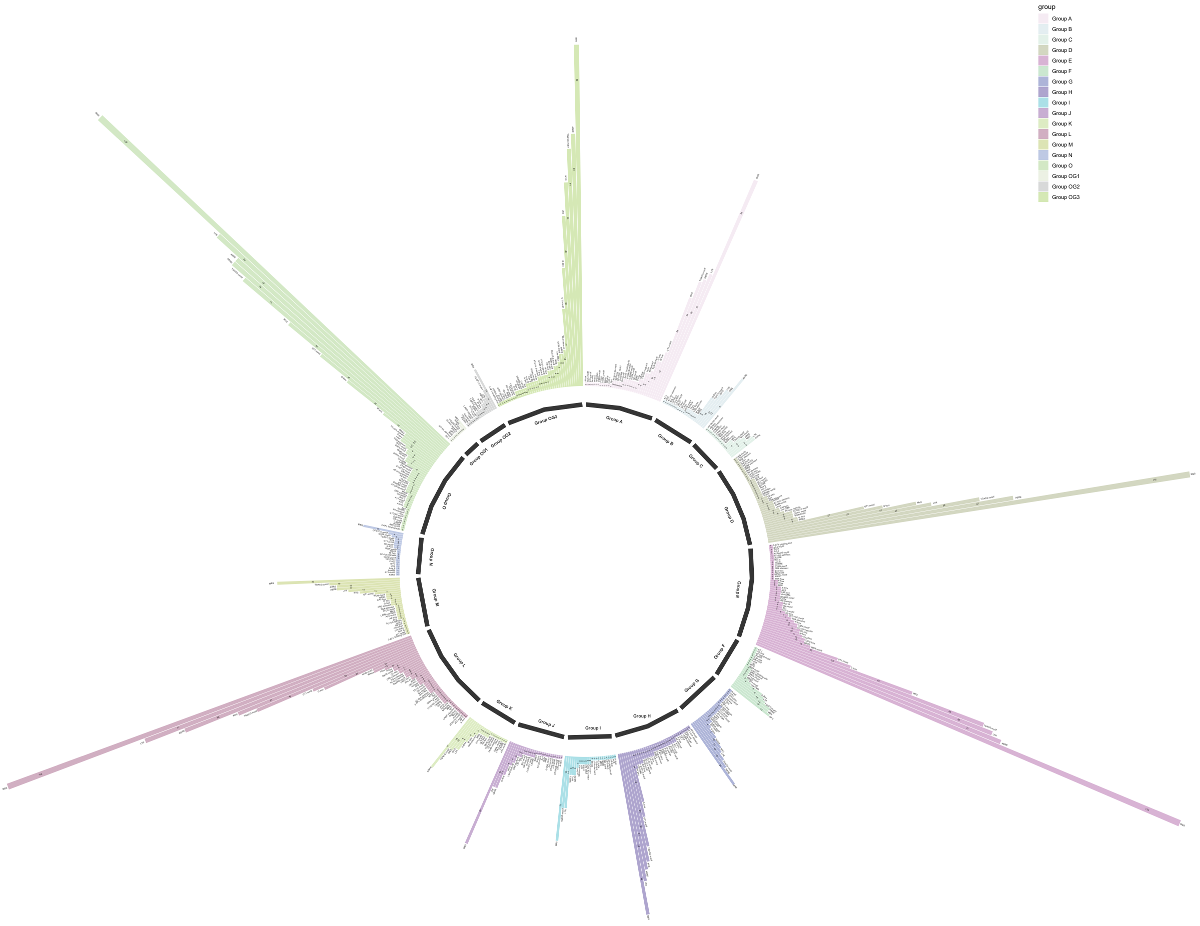
group
Group A
Group B
Group C
Group D
Group E
Group F
Group G
Group H
Group I
Group J
Group K
Group L
Group M
Group N
Group O
Group OG1
Group OG2
Group OG3
